# Eco-friendly synthesis of silver nanoparticles: multifaceted antioxidant, antidiabetic, anticancer, and antimicrobial activities

**DOI:** 10.1038/s41598-025-22154-4

**Published:** 2025-10-27

**Authors:** Nabila G. Elmehalawy, Mahmoud M. M. Zaky, Ahmed M. Eid, Amr Fouda

**Affiliations:** 1 Botany Department, Faculty of Science, Port Said University, Port Said, Egypt; 2 Department of Botany and Microbiology, Faculty of Science, Al-Azhar University, Nasr City, Cairo, 11884 Egypt

**Keywords:** Green synthesis, *Rosmarinus officinalis* extract, Silver nanoparticles, Biomedical and pharmaceutical applications, Multidrug resistant bacteria, Biochemistry, Biotechnology, Drug discovery, Microbiology, Nanoscience and technology

## Abstract

Diabetes, cancer, and multidrug-resistant bacteria are major global health threats, driving the search for novel, safe, and affordable therapeutics. Here, silver nanoparticles (Ag-NPs) were biosynthesized using the aqueous extract of *Rosmarinus officinalis* L. via a sustainable, eco-friendly, and cost-effective green approach. Characterization by UV–Vis, FT-IR, EDX, XRD, TEM, SEM, TGA, DLS, and zeta potential confirmed the formation of well-dispersed, spherical, crystalline nanoparticles with an average size of 60.5 nm, uniform morphology, and high thermal stability. The Ag-NPs displayed concentration-dependent multifunctional activities. Antibacterial assays revealed strong effects against both standard and MDR strains, including *Bacillus subtilis*, *Staphylococcus aureus*, *Pseudomonas aeruginosa*, *Escherichia coli*, and *Klebsiella pneumoniae* (inhibition zones: 11.7–29.7 mm). Potent antioxidant activity was observed with an EC₅₀ of 7.81 µg mL⁻^1^, close to ascorbic acid (3.27 µg mL⁻^1^). Antidiabetic activity reached 85.5% (α-amylase) and 82.6% (α-glucosidase) inhibition at 1000 µg mL⁻^1^, comparable to acarbose (97.5% and 96.3%). Moreover, Ag-NPs showed selective cytotoxicity against MDA and PANC-1 cells (IC₅₀: 177.2 and 115.3 µg mL⁻^1^), with lower toxicity toward Vero and Wi38 normal cells (IC₅₀: 233 and 207 µg mL⁻^1^). These findings highlight the promise of *R. officinalis*–mediated Ag-NPs as multifunctional nanomaterials for biomedical applications.

## Introduction

Antibiotic-resistant bacteria, diabetes, and cancer are among the leading causes of death worldwide due to the health risks they cause and their increasing prevalence over time^1^. In 2019, the estimated number of fatalities caused by antibiotic-resistant bacterial infections ranged between 3.62 and 6.57 million. Of these 3.57 million deaths were associated with resistance to the six most prevalent bacterial infections (*Staphylococcus aureus, Pseudomonas aeruginosa, Escherichia coli, Klebsiella pneumoniae, Acinetobacter baumannii, and Streptococcus pneumonia*); methicillin-resistant *Staphylococcus aureus* alone accounted for nearly 100,000 deaths. This ongoing crisis is primarily driven by the overuse and misuse of antibiotics, particularly their incorrect application, which further contributes to the global burden of antimicrobial resistance^2^. Additionally, in a “One Health” context, the transmission of antibiotic-resistant bacteria (ARB) from food animals can significantly affect both human and animal health. Given the impact of ARB on global health and the urgent need for novel antibiotics, new strategies are being developed to protect and treat PDR (pandrug-resistant), XDR (extensively drug-resistant), and MDR (multidrug-resistant) infections, even though "antibiotics of last resort" are becoming less effective in clinical practice. Several approaches have been explored to address this challenge^3^.

In addition, cancer develops when certain body cells proliferate uncontrollably and spread to other organs. Approximately 10 million people die from cancer each year. Cancer, especially lung cancer, is the second leading cause of mortality globally, accounting for 1.69 million deaths annually. The worldwide economic burden of cancer is projected to reach an astounding $25 trillion^4^. Conventional cancer treatments such as chemotherapy and radiotherapy often cause severe physical and psychological suffering for cancer patients. As a result, nanoparticles (NPs) have emerged as a promising substitute for safer and more effective cancer treatments, including personalized drug delivery, stimuli-responsive drug release, and co-delivery of multiple drugs. Furthermore, in addition to overcoming the shortcomings of traditional cancer treatment, nanoparticles circumvent multidrug resistance. Nanoparticles have been the subject of increasing research in recent years in an effort to discover and create new mechanisms for multidrug resistance^5^. By providing the potential for combined drug therapy and interfering with drug resistance mechanisms, nanomedicines have advanced the fields of targeted drug delivery and tumor multidrug resistance management^6^. The multidisciplinary field of nanotechnology focuses on creating new active compounds at the nanoscale (1–100 nm). Bulk materials lack the fascinating properties of these nanostructures^7^. These special characteristics, which increase their uses in a variety of sectors, include their small size, strong reactivity, a high surface area-to-volume ratio, thermal conductivity, magnetic and optical properties, chemical stability, surface properties, and biocompatibility, thereby expanding their applications in various fields^8,9^.

*Rosmarinus officinalis* L. (Family: Lamiaceae) commonly known as rosemary, has applications in cosmetics and traditional medicine across ancient Egypt, India, China, and Mesopotamia; thus, it is recognized as a medicinal and aromatic evergreen shrub. It has proven effective in treating conditions such as dermatitis, eczema, and acne. The plant is characterized by a range of metabolites, including carnosic acid, betulinic acid, caffeic acid, rosmarinic acid, and various terpenoid compounds. These compounds exhibit notable antibacterial properties against *Staphylococcus aureus* and function as antioxidants, inhibiting UV-induced photoaging^3,10^. Owing to its rich metabolic composition, it represents a promising option for the production of various metals and their oxide nanomaterials.

Nanoparticles are produced by two primary approaches: top-down and bottom-up methodologies. These nanomaterials are generated through physical, chemical, and biological techniques (green techniques). Green techniques rely on using natural substances derived from algae, bacteria, fungi, yeast, actinomycetes, and plants, including herbal sources, to reduce precursors into nanostructures, followed by encapsulation (or capping) to enhance nanoparticle stability^11^. Consequently, green techniques are favored over chemical and physical techniques due to their cost-effectiveness, lack of toxic by-products, environmental safety, simplicity, biocompatibility, sustainability, milder condition requirements, scalability, and varied functionalization by natural metabolites, making them suitable for integration into the medical field. In this regard, the numerous metabolites produced by *Rosmarinus officinalis*, including polysaccharides, phenols, carbohydrates, flavonoids, proteins, alkaloids, and terpenoids, serve as reducing/capping/stabilizing agents. Recently, various metal ions have been reduced by herbal extracts to generate nanoparticles such as iron oxide (Fe), gold (Au), selenium (Se), zinc oxide (ZnO), palladium (Pd), copper oxide (CuO), and silver (Ag)^12–14^.

Silver nanoparticles (Ag-NPs) rank among the most significant nanomaterials and have gained increasing attention across various domains, particularly in the field of medicine, due to their distinctive physicochemical properties^15^. Recently, Ag-NPs have been integrated into a range of biomedical applications including cosmetics, the medical textile sector, surgical instruments, antimicrobial agents, wound healing, drug delivery mechanisms, antioxidant agents, anticancer treatments, cytoprotective effects, anti-inflammatory applications, biofilm inhibition, antiviral treatments, nematocidal and larvicidal agents, nanoparticle-based imaging agents, and disinfectants^16,17^. Therefore, Ag-NPs are considered among the most important nanomaterials in consumer goods^18^. Plants widely used to fabricate Ag-NPs as a green approach and used in varied biomedical applications. For instances, methanolic leaf extract of Bauhinia racemosa was utilized for Ag-NPs which exhibit promising antimicrobial activity against prokaryotes and eukaryotes species^19^. Also, the aqueous extract of desert truffles, *Terfezia claveryi,* was used for green synthesis of Ag-NPs have cytotoxic effect on breast cancer cell, MCF7^20^. Moreover, the seed extract of *Phoenix dactylifera* utilized for Ag-NPs with antibacterial, antibiofilm, and in-vitro cytotoxicity^21^**.**

A limited number of studies have employed *R. officinalis* extract for the biosynthesis of Ag-NPs. Most of these studies focused only on one or two functionalities. For example, Ghaedi et al.^10^ used the leaf water extract of *R. officinalis* for the synthesis of Ag-NPs that acted as antimicrobial agents against selected test organisms. Additionally, Sganzerla et al.^22^ utilized *R. officinalis* oil to create Ag-NPs, which were integrated into biopolymers for antioxidant and antimicrobial functions. The present study differs from these prior investigations by analyzing the water extract components of *R. officinalis*, optimizing the biosynthesis process by examining the influence of various environmental factors, and expanding the applications to include, in addition to antimicrobial properties against selected test organisms, multidrug-resistant strains, antidiabetic activity, antioxidant, and anticancer effects.

Therefore, the present research focused on the eco-friendly synthesis of Ag-NPs using *R. officinalis* extract and the exploration of their potential biological activities, considering the application of phyto-nanotechnology for the identification of new nanoparticles with potential therapeutic uses. The optical properties, chemical composition, crystalline structure, morphological characteristics, thermal stability, hydrodynamic size, and surface charge of the biogenic Ag-NPs were evaluated. The in vitro cytotoxic studies, antioxidant, antidiabetic, and antibacterial properties against specific microbial strains and multidrug-resistant strains, were also assessed.

## Materials and methods

### Chemicals, reagents, and plant samples used

All chemicals and reagents used in the current investigation were analytical grades collected from the Sigma Aldrich Office, Cairo, Egypt.

#### Bacterial sٍtrains

Antibacterial activity was evaluated against standard test organisms (*Bacillus subtilis* (ATCC-6633)*, Staphylococcus aureus (*ATCC-6538), *E. coli* (ATCC-8739), and *Pseudomonas aeruginosa* (ATCC-9027) as well as against multidrug-resistant bacterial strains (MDR). The five tested MDR strains were obtained courteously from the Universal Cairo Hospitals Staff, Egypt, and identified as *E. coli* (three strains isolated from urine (*E. coli*-1), sputum (*E. coli*-2), and wound (*E. coli*-3) samples) and *Klebsiella pneumonia* (two strains isolated from wound and urine samples). The isolation was conducted with approval from the Port Said University Institutional Review Board (IRB), Faculty of Science (2023–2024), the informed consent was obtained from all patients. The identification of the obtained MDR bacterial strains was conducted using Vitek-2. The *E. coli* strains were recognized as ESBL (Extended-Spectrum β-lactamase), whereas one strain of *K. pneumonia* was recognized as XDR (Extensively Drug-Resistant) and identified as *K. pneumonia*-1, while the second strain was recognized as ESBL and identified as *K. pneumonia*-2. The MDR strains were resistant to aminoglycosides (amikacin and gentamicin), cephalosporins (ceftriaxone and cefotaxime), fluoroquinolones (ciprofloxacin and levofloxacin), and carbapenems (meropenem, imipenem).

#### Cell lines

The biocompatibility of biosynthesized Ag-NPs was assessed against two normal cell lines, namely Wi38 (lung fibroblast) and Vero (kidneys epithelial cells), while their anticancer activity was evaluated against MDA (breast cancer cells) and PANC-1 (pancreatic cancer cells). The normal and cancer cells were obtained from VACSERA, Cairo, Egypt an accredited cell culture center. All experimental methods conducted in the present research were performed in accordance with applicable institutional, national, and international standards and regulations. Additionally, we affirm that this research did not include human subjects or human-derived materials. The chosen cell lines used in this study were acquired commercially and did not necessitate ethical clearance.

#### Plant samples

The green synthesis of Ag-NPs was accomplished by employing an aqueous extract of *Rosmarinus officinalis* L. leaves gathered from cultivated soil in Saint Catherine (28°33′25. 4″ N; 33°56′52. 2″ E), located in South Sinai Governorate, Egypt. Our research adhered to institutional, national, and international regulations and guidelines. *R. officinalis* is prevalent globally and does not require permits or licenses, as the species we are utilizing is a cosmopolitan crop that is neither endangered nor endemic according to IUCN. The sampling was conducted with authorization (number: SK21/2023) from the local agricultural office in the governorate. The botanical identification of *Rosmarinus officinalis* was verified at the Herbarium of the Botany Department, Faculty of Science, Port Said University, Egypt. A voucher specimen has been deposited in the same herbarium under the registration code *R. officinalis* (1_Ro) for future reference. The harvested leaves were stored in sterilized polyethylene bags prior to being transported to the laboratory in an icebox.

### Preparation of *R. officinalis* aqueous extract

The collected leaves of *R. officinalis* were washed twice with distilled H_2_O (dH_2_O) to remove debris and remained for two days for drying at 37°. After that, the leaves were ground to powder and 10 g of it in 100 mL dH_2_O for 1 h on a magnetic stirrer at 50°C, followed by centrifugation of the mixture and collection of clear supernatants (aqueous extract), which was used to synthesize Ag-NPs.

### Analysis of the *R. officinalis* aqueous extract

The metabolic composition of the plant aqueous extract, which served as a reducing/capping/stabilizing agent, was examined using Gas Chromatography-Mass Spectrometry (GC–MS) analysis (Santa Clara, Agilent Technologies, USA). In this analysis, gas chromatography (7890-B), detector of the mass spectrometer (5977A), HP-5MS as GC column (30 m × 0.25 mm), the thickness of the film (0.25 m), and hydrogen as a gas carrier (1 mL/min flow rate) were used. The analysis was achieved at varied temperature as follows: 50°C for 1 min., an increase of 5°C per minute up to 100 °C, and an increase of 10°C per minute up to 300°C for 5 min. The injection occurred at 250 °C, while the detector temperature was set to 260°C, and the energy for electron ionization was maintained at 70 eV 94. The components of the plant aqueous extract were identified by matching with the NIST and Wiley Library^23^.

### Ag-NPs biosynthesis

For the synthesis of Ag-NPs, 80 mL of aqueous extract from *R. officinalis* leaves was mixed with 20 mL of deionized water (dH_2_O) that contained AgNO_3_ (metal precursor) to achieve a final concentration of 1 mM. The pH of the resulting mixture was 8 and it was kept under stirring conditions (150 *rpm*) for one hour. To verify the complete reduction of AgNO_3_, the solution was stored at room temperature in the dark for 24 h^10^. To collect the Ag-NPs, the resulting mixture was placed in an air oven at 70 °C to evaporate the liquid, followed by the collection of the residue, which was washed twice with deionized water (dH_2_O) to eliminate any impurities before being calcined for 2 h at 200 °C. The yellowish-brown absorbance was recorded in the range of 200–800 nm using UV–Vis spectroscopy (JENWAY 6305) to identify the maximum surface plasmon resonance (SPR) of the synthesized Ag-NPs^24^.

### Optimization of biosynthesis conditions

To obtain the highest yield of Ag-NPs resulting from the transformation of the metal precursor into the nanostructure, the biosynthesis conditions were optimized. The determination of optimal conditions was achieved by measuring the absorbance of newly formed color at maximum SPR (λ_max_ = 415 nm). The parameters examined included pH value (in the ranges of 6–11), metal precursor (1–4 mM), plant extract concentration (9:1, 1:9, 2:8, 8:2, and 1:1 of plant extract: dH_2_O with AgNO_3_), mixing time (contact time) between metal and plant extract (1–3 h), and incubation temperature (35–65 °C).

### Green synthesized Ag-NP characterizations

The plant-synthesized Ag-NPs under optimal conditions underwent physicochemical characterization, including chemical composition (Fourier transform infrared, FT-IR; and energy dispersive X-ray, EDX), crystalline structure (X-ray diffraction, XRD), morphological characteristics (Transmission electron microscopy, TEM; and scanning electron microscopy, SEM), thermal stability (thermogravimetric, TGA), hydrodynamic size (dynamic light scattering, DLS), and surface charge (zeta potential). For FT-IR analysis, 10 mg of nano-Ag (or a few drops of plant aqueous extract) and KBr were mixed, compressed to form a disc, and scanned in the ranges of between 400 to 4000 cm^–1^^25^. For XRD, PANalytical-X’Pert-Pro-MRD with a current of 30 mA, a voltage of 40 kV, and an X-ray source (CuKα) with λmax 1.54 Å was used to investigate the crystalline structure. The analysis was achieved in the 2theta (2θ) ranges of 10°–80°. Moreover, XRD analysis gives more information about crystallite size through calculation using Debye–Scherrer’s equation^26^:

1 $$A{ = }\frac{{(0.94) \times {(1}{\text{.54)}}}}{\beta \cos \theta }$$

where A is the average size of the crystal, 0.94 is the Debye–Scherrer’s constant, 1.54 is the X-ray λmax, β is the half-maximum of the diffraction peak, and θ is the Bragg’s diffraction angle.

The TEM and SEM analyses were used to investigate the shape, size, and surface characteristics of synthesized Ag-NPs. In TEM (LTD 1010, JEOL, Japan) analysis, the synthesized Ag-NPs were sonicated with ultra-pure H_2_O followed by adding few drops on the TEM grid surface before subjected to scanning^27^. In SEM (JSM 6360, JEOL) analysis, a few drops of Ag-NPs suspended solution were added to the SEM carbon-coated Cu grid and subjected to drying using a mercury lamp for 5 min before analysis^10^. The elemental mapping of synthesized NPs was assessed by EDX (Thermo-Fisher Scientific, USA) analysis.

The hydrodynamic sizes and their distribution in colloidal solutions were analyzed using DLS. For this analysis, the high-purity water was used to dissolve the plant-synthesized NPs to avoid shadow formation and anomalous peaks during NP scattering. Additionally, the surface charge of the synthesized structure was assessed by zeta potential (ζ) value using a Malvern Zeta-sizer (Nano-ZS, Malvern, UK).

The thermal stability of Ag-NPs was assessed using TGA (SDT Q600, USA) analysis in the presence of atmosphere nitrogen (20 mL/min) with a heating rate of 15°C per minute and temperature ranges of 30–900 °C^28^.

### Biomedical applications of the green synthesized Ag-NPs

#### Antibacterial activity

The antibacterial activity of rosemary-mediated synthesized Ag-NPs was evaluated against coded test organisms and MDR bacterial strains by the agar well diffusion method. For this assay, the dried surface of Mueller–Hinton agar media was streaked in three directions. The bacterial suspension was adjusted to 0.5 McFarland standard (approximately 1–2 × 10^8^ CFU mL^–1^) prior to inoculation. Wells were performed using a sterile cork borer (0.6 mm). After that, 100 µL of nano suspension (dissolved in DMSO) with a concentration of 300 µg mL^–1^ was added to each well and kept in the refrigerator for one hour before being incubated at 37 °C for 24 h. After the incubation period, the zones of inhibition were measured by millimeter (mm) at the point where significant growth reduction was observed^29^. For MIC value determination, the activity of double-fold Ag-NPs concentrations (200–12.5 µg mL^–1^) was analyzed as mentioned above. The MIC value is the lowest concentration causing an inhibition zone. The activity of negative control (DMSO) and positive control (Gentamycin) was assayed under the same condition. The experiment was conducted in triplicate.

#### Antidiabetic activity

The inhibitions of two enzymes, α-amylase and α-glucosidase, *vs.* positive control (acarbose) were used as a marker for the antidiabetic activity of nano-Ag.

*Inhibition of α-amylase activity*: Different concentrations (1000–1.95 µg mL^–1^) of AgNO_3_ nanostructure and acarbose were prepared in separate test tubes containing the following mixtures: 0.02 M phosphate buffer (pH = 6.9) + 500 µL of α-amylase and mixed well before being incubated at 37 °C for 10 min. Afterwards, 500 µL of 1% starch solution was added to each tube and incubated for another 10 min. For the α-amylase inhibition assay, 1 mL of DNS (3,5-dinitrosalicylic acid) was added to each tube and incubated for 15 min at 60 °C in a water bath, cooled, and mixed with 10 mL of dH_2_O. The absorbance of the formed color was measured spectrophotometrically at 540 nm, and α-amylase inhibition % was calculated as follows^30^;

2 $${\text{Inhibition}}\;\alpha {\text{-}} {\text{amylase}}\% = \frac{{{\text{A}} - {\text{B}}}}{{\text{A}}} \times 100$$

where A and B are the absorbance of control and treatment, respectively.

**Inhibition of α-glucosidase activity**: 100 µL of Ag-NPs or acarbose concentration was mixed with 150 µL sodium phosphate buffer (0.1 M) and 1 U of α-glucosidase enzyme in a test tube, followed by incubation at 37°C for 10 min. After that, 50 µL of 2mM of *p*-nitrophenyl-D-glucopyranoside was added to tube and incubated for 20 min at 35 ± 2°C. To stop the reaction, 50 µL of sodium carbonate (0.1 M) was added, and the absorbance of the formed color was assessed at 405 nm to calculate the α-glucosidase inhibition % using the above Equation number 2.

#### Antioxidant activity

The antioxidant activity of plant-mediated biogenic Ag-NPs were evaluated via DPPH scavenging activity. Milli Q-H_2_O (high pure H_2_O) was used to dissolve the nano-Ag at different concentrations separately (1000 to 1.95 µg mL^−1^) where methanol was used to dissolve the DPPH. For the DPPH assay, 1.0 mL of each concentration was mixed with 1.0 mL of DPPH and 450 µL of Tris–HCl buffer (pH = 7.4), mixed well, and incubated in a dark condition at 37°C for 30 min under a shaking condition (150 *rpm*). Ascorbic acid at the same concentrations was used as a positive control, whereas the negative control was a test tube containing all components except tested substances (Ag-NPs or ascorbic acid). Spectrophotometrically, the scavenging activity was measured at 517 nm, and the percentages were calculated as follows^31^;

3 $${\text{DPPH}}\, {\text{scavenging}} \% = \frac{{{\text{Control}} \,{\text{absorbance}} - {\text{Treatment}}\,{\text{absorbance}}}}{{\text{Control absorbance}}} \times 100$$

#### Anticancer activity

The MTT assay was conducted to investigate the cytotoxic properties of *Rosmarinus officinalis*-derived Ag-NPs against two normal cell lines, Wi38 (human lung fibroblasts) and Vero (monkey’s kidney epithelial cells) and two cancer cell lines, MDA (human breast adenocarcinoma) and PANC-1 (human pancreatic epithelioid carcinoma) (VACSERA, Egypt). Briefly, 100 μL of each cell line (1 × 10^5^ cells/well) was inoculated into 96-well tissue culture plates and incubated at 37°C for 24 h in a CO_2_ incubator (5%). Once the monolayer cell sheet was formed, it was rinsed twice and then 100 μL of maintenance media (RPMI with 2% serum) was added before treating the cloned cells with double-fold dilutions of nano-Ag (1000–31.25 μg mL^–1^) and incubated again for 48 h. Control samples were prepared in triplicate wells without Ag-NPs. Following an incubation period, the maintenance media was discarded, and 50 μL of MTT solution (5 mg mL^–1^) was introduced into each well; the plates were agitated (5 min/150 *rpm*) and incubated for 4 h at 37 °C. The MTT solution was substituted with 200 µL of 10% DMSO, and the plates were shaken to fully dissolve the produced formazan crystals^32^. The toxicological effects of silver NPs were evaluated by determining OD with an ELISA reader (λ = 570 nm), and the cell viability percentages were calculated as follows:

4 $$\% {\text{of}} \,{\text{cell}} \,{\text{viability}} = \frac{{treated{\text{ sample absorbance}}}}{{Control{\text{ absorbance}}}} \times 100$$

Morphological changes in cell lines induced by silver nanoparticles were investigated by inverted microscopy (Nikon, ECLIPSE Ts2, Shinjuku, Tokyo, Japan).

### Data analysis

Data in this study were examined using SPSS (version 18, USA). The subsequent analyses were conducted: the Shapiro–Wilk test, the equal variance test, ANOVA, and the post-hoc test.

## Results and discussion

### *R. officinalis* extract analysis and Ag-NPs synthesis

**Fig. 1:**
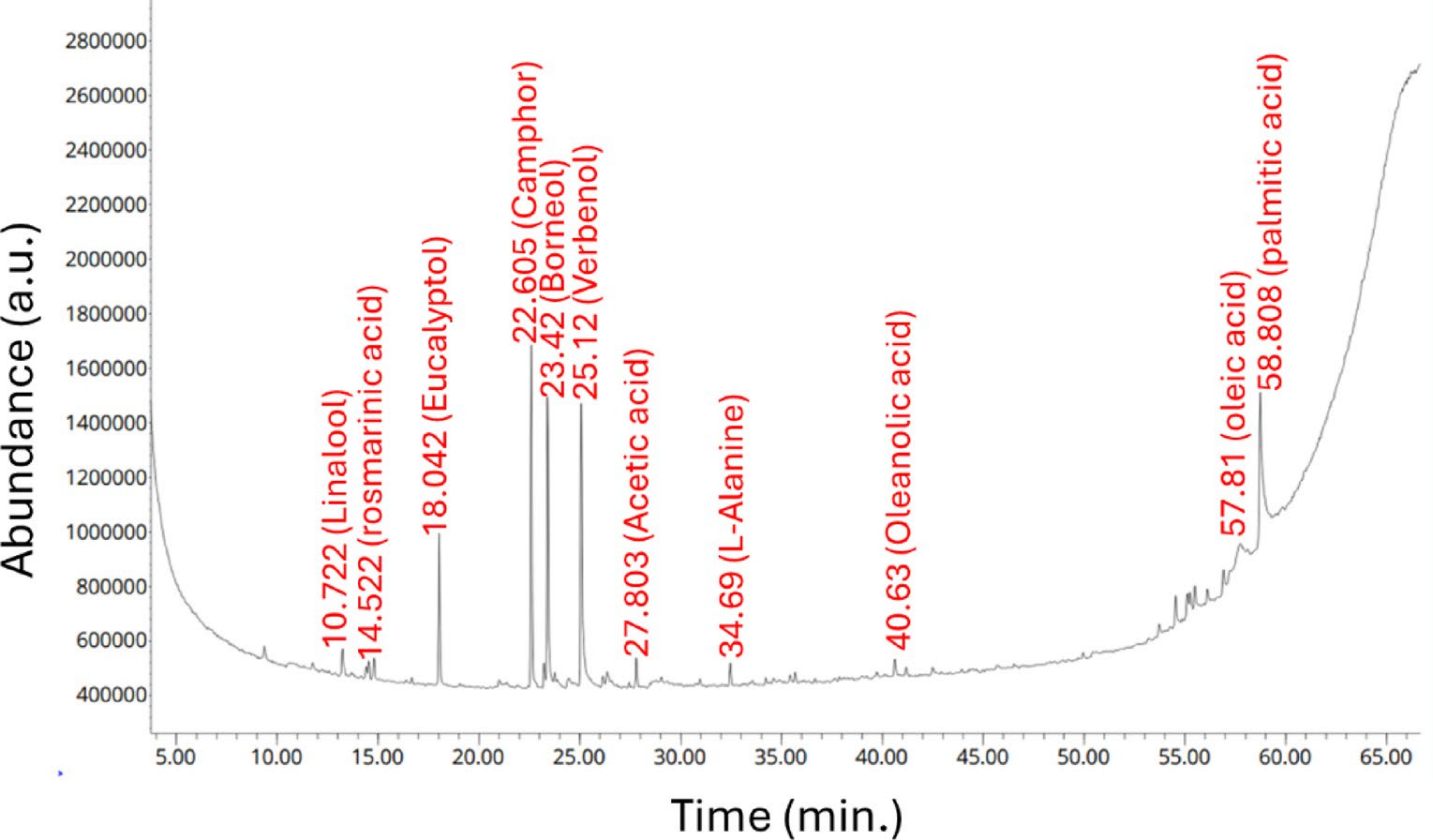
GC–MS analysis of *R. officinalis* extract reveals peaks corresponding to several active metabolites at designated retention times.

The use of plant extracts for NP synthesis as an eco-friendly method offers benefits in addressing the issues associated with physical and chemical techniques^33^. This eco-friendly method is advantageous for its rapidity, cost-effectiveness, avoidance of potential infections that can arise from using microorganisms for biosynthesis, high yield, biocompatibility, and significant production of metabolites from plants that act as reducing agents, providing specific shapes and sizes, and improving NP stability^34^. Nonetheless, the fabrication of green NPs using plant extracts relies on their constituents, such as terpenoids, flavonoids, alkaloids, and so forth, which serve as reducing agents for metal precursors in the formation of nanostructures. Consequently, determining the components of *R. officinalis* extract poses the primary challenge in understanding the bio-fabrication process. In this context, GC–MS (Fig. 1) was employed to identify the components of *R. officinalis* extract. The results revealed that eucalyptol was detected at a retention time (RT) of 18.042 with a percentage area of 0.36%. This compound was identified as cineole, categorizing it as a monoterpenoid. Additionally, Borneol (camphor), was found at RT 22.6 and 23.4 with percentage areas of 0.21 and 0.32%, respectively, and was classified within terpenoids and cyclic ketones. Furthermore, acetic acid, rosmarinic acid, methional, linalool, oleic acid, palmitic acid, and taurolidine were identified in the GC–MS analysis. Aside from reducing metal precursors, these compounds serve as capping agents that enhance stability and improve the biological activities of the synthesized nanostructures. Recently, phytochemical analyses of *R. officinalis* extracts have confirmed the presence of diverse metabolites, including flavonoids, essential oils, phenolic compounds, rosmarinic acid, ursolic acid, terpenoids, and betulinic acid^35^.

**Fig. 2:**
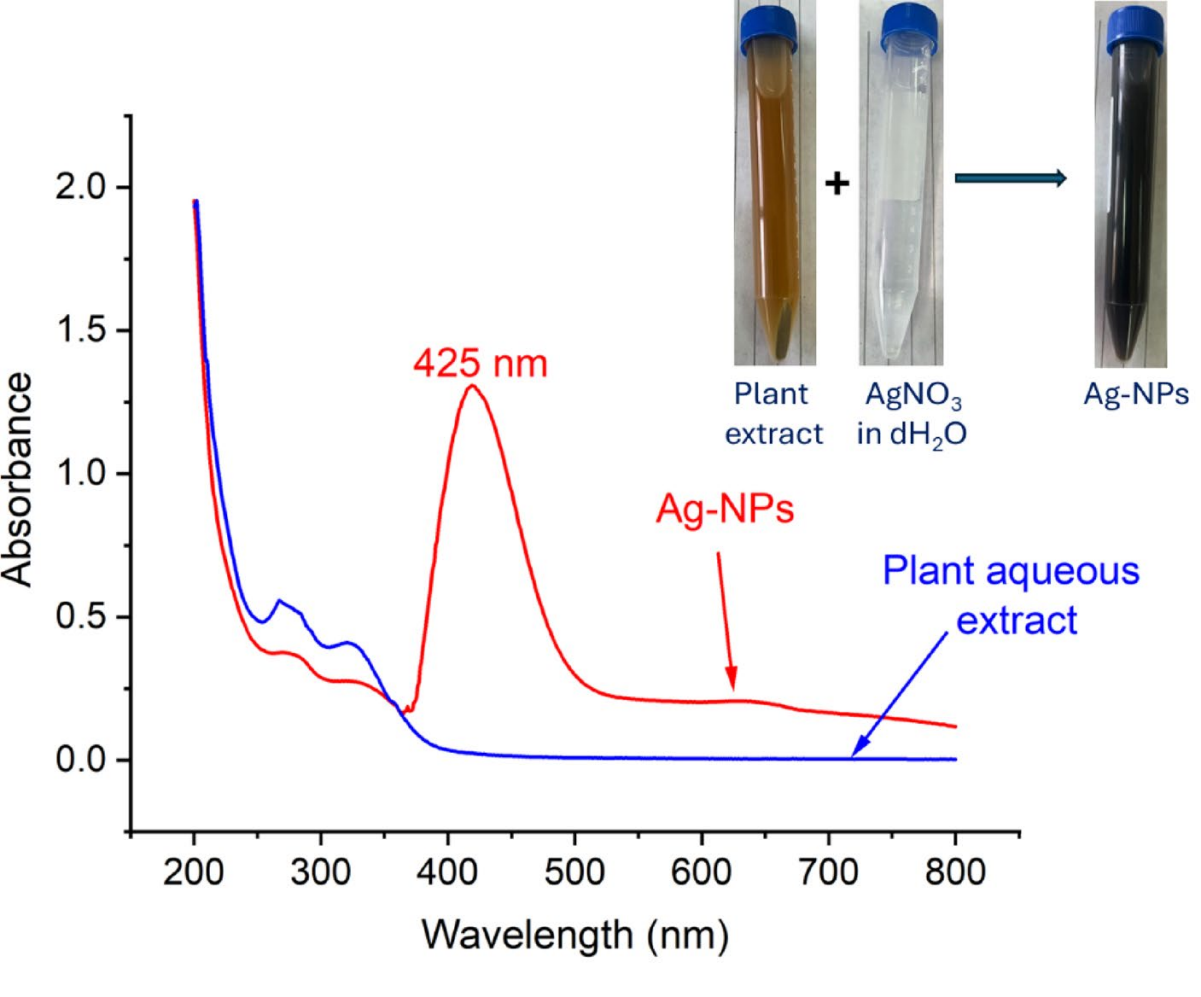
UV spectrum of green synthesized Ag-NPs (peak SPR at 425 nm) and *R. officinalis* aqueous extract.

In the present study, the effective transformation of Ag^+^ into Ag^0^ by the interaction of *R. officinalis* leaf aqueous extract was validated by a color change from colorless to yellowish-brown. UV–vis analysis was employed to evaluate the color intensity to identify the maximum surface plasmon resonance (SPR) in relation to the plant extract. As demonstrated, the occurrence of SPR at a wavelength of 425 nm confirms the formation of Ag-NPs (Fig. 2). Likewise, the SPR of *Zingiber officinale*-Ag-NPs was found at 425 nm, and the authors established that the peak at this wavelength was associated with a spherical shape^36^. Dong and colleagues noted that the typical SPR resulting from the reduction of Ag^+^ to form Ag^0^ and the creation of Ag-NPs occur within the ranges of 400–450 nm^37^. Consistent with this observation, the SPR peak of rosemary-Ag-NPs was detected at 450 nm^10^. Additionally, the nano-Ag-SPR generated by the fruit extract of *Viburnum opulus* L. was recorded at a wavelength of 415 nm^28^. Conversely, the SPR peak of Ag-NPs produced by various microbial domains (bacteria, fungi, and actinomycetes) appeared as a broad peak within the range of 420–430 nm^38^. The variations in the SPR peak value might be attributed to the different metabolites present among the various biological entities acting as reducing/capping agents^39^. The UV spectrum of *R. officinalis* extract displayed two peaks within the ranges of 250–350 nm, which are linked to phenolic and flavonoid compounds such as rosmarinic acid, in alignment with GC–MS analysis.

### Optimizations of Ag-NP synthesis

The optimization of environmental production conditions plays a crucial role not only in boosting productivity but also in positively influencing size, shape, stability, and functional characteristics that are appropriate for integrating NPs into various applications^34^. In this context, the influence of AgNO_3_ concentration, pH value, contact time, temperature, and plant extract concentration was examined with respect to color intensity, which signifies high Ag^+^ reduction and consequently high productivity at a peak SPR of 425 nm.

**Fig. 3:**
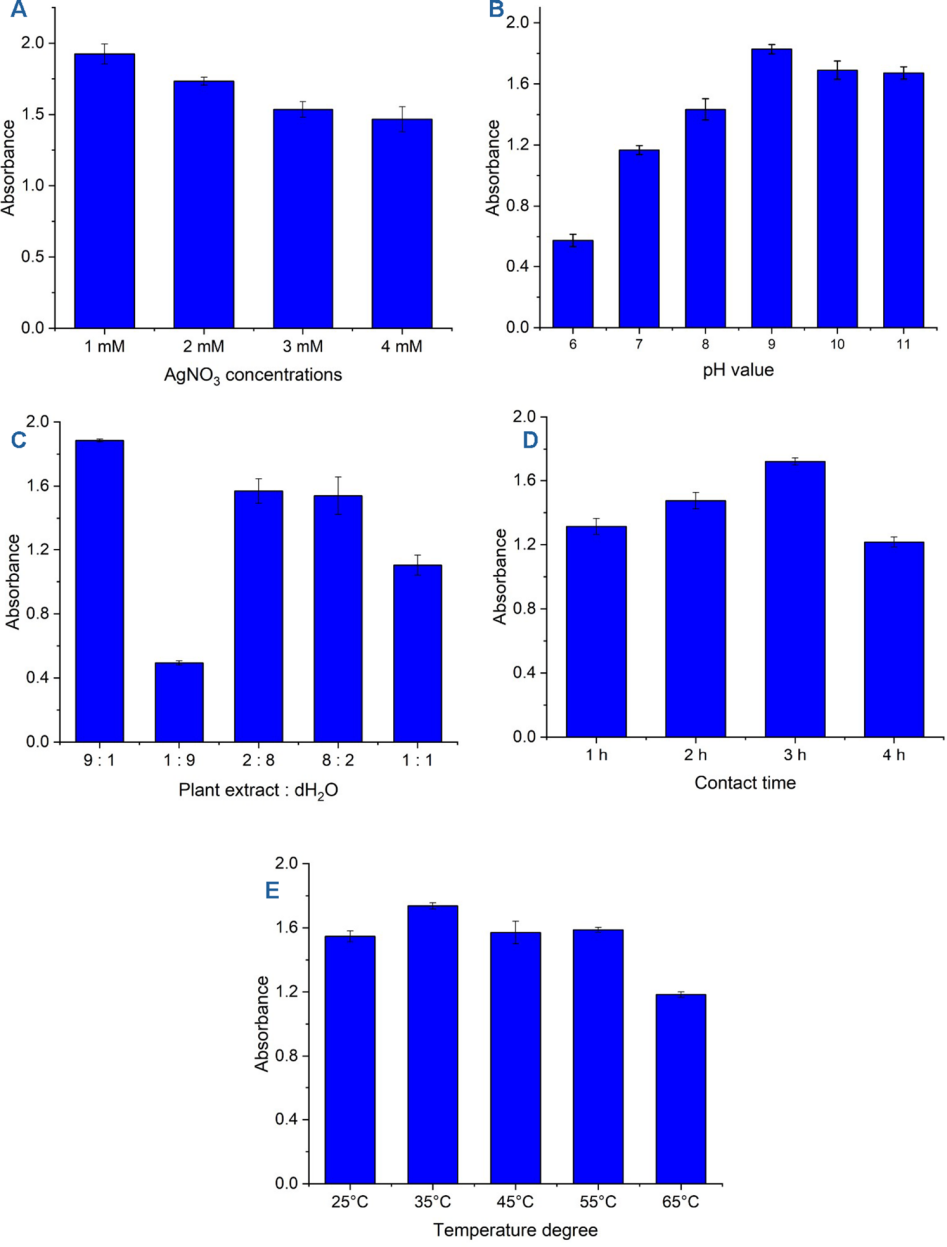
Optimization of various parameters for Ag-NPs synthesis. (**A**) impact of different AgNO_3_ concentrations, (**B**) effect of pH values, (**C**) comparison of plant extract and dH_2_O, (**D**) influence of contact time on Ag-NPs synthesis, and (**E**) effect of incubation temperature on the biosynthesis process.

Concerning metal precursor concentration, the data obtained indicated that the most intense yellowish-brown color was achieved at an AgNO_3_ concentration of 1 mM, which then decreased with varying metal concentrations (Fig. 3A). Consistent with color intensity, the peak absorption (1.92) was attained at 1 mM AgNO_3_. This absorption decreased to 1.73, 1.53, and 1.46 at 2, 3, and 4 mM, respectively (Fig. 3A). The data obtained demonstrated that an AgNO_3_ concentration of 1 mM was optimal for complete reduction by plant extract to generate Ag-NPs. Likewise, the ideal concentration of AgNO_3_ for the synthesis of Ag-NPs using the water extract of *Azadirachta indica* leaves was found to be 1 mM, as indicated by changes in color and maximum absorption value compared to 2, 3, and 4 mM^12^. The declining absorption at higher metal concentrations could be attributed to the aggregation of formed NPs, as plant biomolecules might be insufficient to provide protection, resulting in larger sizes and thus lower absorption values^40^.

Conversely, the reduction process, dispersion, and stability of NPs are influenced by the pH values through their effects on the charges of plant metabolites and metal ions. Under acidic conditions (low pH), the formation of NPs is hindered due to competing reactions between excessive protons and Ag^+^. However, at higher pH levels, the reduction process is enhanced, but may lead to particle aggregation, producing larger sizes^41^. Therefore, the optimization of pH levels is a crucial factor during green synthesis. In this study, the effect of varying pH values (6–11) on Ag-NPs synthesis using rosemary leaves extract was analyzed (Fig. 3B). The most significant color change and absorption rate (1.82) occurred at pH 9. At pH 6, a pale yellowish-brown color was noted with the lowest absorption value (0.575). The color intensity elevated with increasing pH, peaking at pH 9, before decreasing again past this point. The findings demonstrated a significant difference in absorption rates at pH 7 (1.16) compared to pH 8 (1.43), while there was no significant difference between absorption values at pH 10 (1.68) and pH 11 (1.67). This indicates that alkaline conditions, particularly at pH 9, were the optimal pH value for Ag-NP synthesis using rosemary, in contrast to acidic or neutral conditions.

The selection of the optimal plant aqueous extract versus dH_2_O influences the interaction of the metal precursor and enhances the capping agents, resulting in the long-term stability of formed nanostructures^8^. In this study, various concentration ratios of rosemary extract versus dH_2_O (that included AgNO_3_) were prepared as follows: 9:1, 1:9, 2:8, 8:2, and 1:1 mL (Fig. 3C). Data analysis indicated that the lowest absorption rate (0.493), along with the minimal color change, was reached at a plant extract concentration versus dH_2_O with a ratio of 1:9 mL, due to the lowest amount of plant metabolites that reduced AgNO_3_ to generate NPs. Conversely, the highest yellowish-brown coloration, corresponding to an absorption rate (1.88), was noted when dH_2_O (containing AgNO_3_) interacted with 9 mL of plant extract.

To determine the optimal contact time, rosemary leaf extract at optimal concentration was combined with 1 mM AgNO_3_ at pH 9 for 1, 2, 3, and 4 h. The data analysis demonstrated that the yellowish-brown color, indicating maximum AgNO_3_ reduction, was achieved after three hours of mixing (Fig. 3D). Following one hour, the degree of color change was faint due to the gradual conversion of AgNO_3_, leading to a minimal absorption rate (1.31). As the contact time increased, the reduction process accelerated, resulting in a deeper yellowish-brown color and achieving the maximum absorption rate (1.72) after three hours. Beyond this point, Ag-NPs began to agglomerate, causing a decrease in the absorption rate, which dropped to 1.21 at a contact time of four hours. Previously published studies support the present findings regarding the optimal contact time for the reduction of metal precursors to form nanostructures^42,43^.

Temperature is a crucial factor influencing the kinetic reduction process and NP yield rates. At lower temperatures, the reaction rate is slower, although this allows for better size control. In contrast, the reduction rate rises at higher temperatures but may cause agglomeration of the synthesized particles, resulting in a reduced SPR value. Therefore, moderate temperatures are preferred for producing smaller, more dispersed, and stable NPs^15^. Here, under all the aforementioned optimal conditions, the reaction process was conducted at various incubation temperatures of 25, 35, 45, 55, and 56 °C (Fig. 3E). The data revealed that the maximum absorption rate (1.73) was achieved at an incubation temperature of 35°C, owing to the higher activity of bioactive reducing molecules. In contrast to the current results, the optimal temperature for forming nano-Ag by leaves in Neem water extract was 60°C, which yielded the highest absorption value, while particle agglomeration above this temperature (at 70°C) led to a decrease in the absorption rate^12^.

### Ag-NPs characterization

**Fig. 4:**
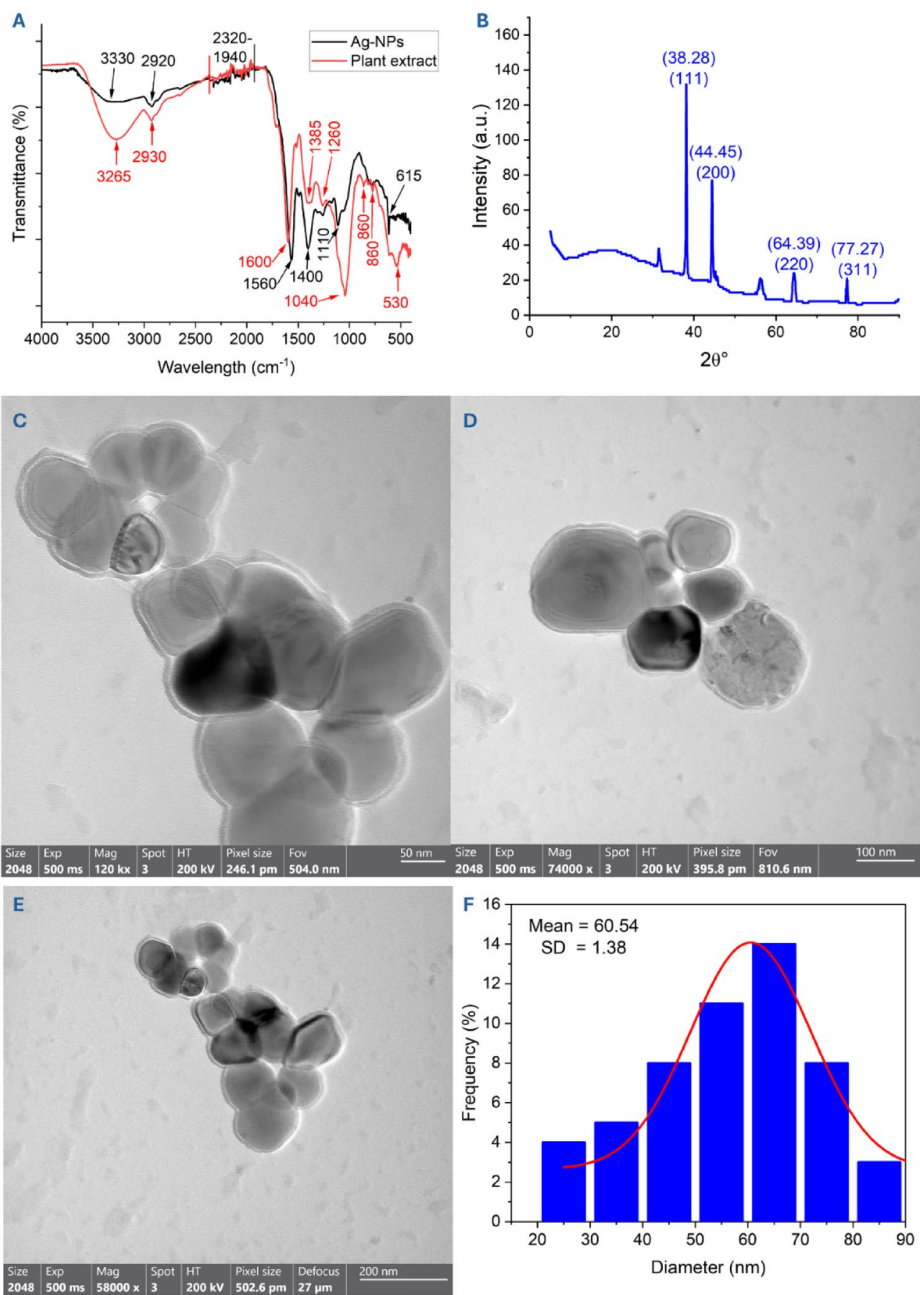
Characterization of rosemary-mediated synthesis of NPs. (**A**) FT-IR for synthesized Ag-NPs compared to plant extract displaying various functional groups, (**B**) XRD analysis confirmed the crystalline characteristics of biogenic Ag-NPs, (**C**, **D**, and **E**) TEM images at varying magnification levels, 50, 100, and 200 respectively illustrating spherical forms, and (**F**) presents the size distribution histogram.

The characteristic groups for various bioactive compounds in the plant extract, including amines, amino acids, carbohydrates, polysaccharides, and proteins, and their roles in the reduction process for nano-Ag synthesis were determined by FT-IR (Fig. 4A, Table 1). The broad stretching peak of primary amines (N–H) overlapping with the hydroxyl (O–H) group was observed as a broad peak in rosemary extracts at a wavelength of 3265 cm^–1^^44^. Following Ag-NP fabrication, a new weak peak at a wavelength of 3330 cm^–1^ was noted. Additionally, the aliphatic C–H stretching peak appeared at a wavelength of 2930 cm^–1^ (plant extract) or 2920 cm^–1^ (Ag-NPs)^45^. The intensity and emergence or disappearance of peaks between 2320 to 1940 cm^–1^ may be linked to aromatic C–H bending, allene C=C=C stretching, or alkyne C≡C stretching^45,46^. The IR spectrum of the plant extract displaying a strong peak at 1600 cm^–1^ (shifted to 1560 cm^–1^ after Ag-NPs synthesis) suggests the presence of C=O stretching of ketones or amides or N–H bending of secondary amines^47^. A peak at 1385 cm^–1^, which shifted to 1400 cm^–1^ after Ag-NPs synthesis, indicates the O–H bending carboxylic acid group^48^. A strong C–O stretching at a wavelength of 1040 cm^–1^ corresponds to the vinyl ether, which shifted to medium 1110 cm^–1^ upon Ag-NPs formation for C–O stretching of secondary alcohols^49^. Conversely, the presence, absence, or variation in peak intensity between plant extract and Ag-NPs in the ranges of 900–500 cm^–1^ indicates halogen compound groups^42^. Notably, the peak at 615 cm^–1^ confirms the existence of Ag at a nanoscale structure as previously reported^50^. The IR chart suggests that the bioactive molecules, like hydroxyl, carbonyl amides, and amines, found in the plant extract remain attached or coat the surface of Ag-NPs, enhancing their stability.

**Table 1:** Key FTIR peaks of *Rosmarinus officinalis* extract and Ag-NPs with their corresponding functional groups.

Plant extract (cm⁻^1^)	Ag-NPs (cm⁻^1^)	Tentative assignment (vibration)	Functional groups likely involved and reference (s)
3265	3330	ν(O–H), ν(N–H) (H-bonded)	Phenolic –OH, amines/proteins (reducing & capping)^44^
2930	2920	ν(C–H) of aliphatic –CH₂/–CH₃	Lipids/terpenes; organic matrix (capping)^45^
2320–1940	2320–1940 (weak/overtones)	Combination/overtones; possible ν(C≡C)/ν(C≡N)	Minor contributions from alkynes/nitriles^45,46^
1600	~ 1600	ν(C=O) Amide I/ν(C=C) aromatic	Proteins/polyphenols binding to Ag surface^47^
1385–1260	1385–1260	δ(O–H), ν(C–N)	Phenols (O–H bend), amines (C–N stretch)–capping^49^
1040	1040	ν(C–O)	Alcohols, esters, carbohydrates (reducing/capping)^49^
860–680	860–680	Aromatic δ(C–H) out-of-plane	Aromatic rings of polyphenols (capping)^42^
—	615–530	M–O (metal–ligand)	Ag–O/interaction of phytochemicals with AgNP surface^50^

The crystallography of nano-Ag synthesized from plants was examined via X-ray diffraction. The XRD chart shows that the various Bragg diffraction peaks of (111), (200), (220), and (311) emerged at 2θ° of 38. 28°, 44. 45°, 64. 39°, and 77. 27°, respectively (Fig. 4B). The results confirmed that the produced Ag-NPs were crystalline with a face-centered cubic structure based on the JCPDS file number 04-0783^28^. The detection of some peaks in the XRD chart indicates slight impurities associated with the scattering of plant-coated agents. The Debye–Scherrer’s equation was utilized to determine the average crystallite size of nano-Ag based on XRD analysis. Analysis of the data revealed that the average Ag-NP crystallite size was 32 nm. In a similar manner, the average nano-Ag crystallite size generated using *Eugenia roxburghii* aqueous extract was 35 nm^9^, whereas those created by *Morinda lucida* exhibited a crystallite size of 8.8 nm calculated using the Debye–Scherrer’s equation^51^.

The dimensions and configuration of the NPs formed by rosemary aqueous extract were examined using transmission electron microscopy. In this case, the synthesized Ag-NPs were spherical and evenly distributed without agglomeration, with sizes ranging from 20 to 90 nm and an average of 60.5 ± 1.38 (Fig. 4C–F). Likewise, spherical and well-dispersed Ag-NPs were produced from *Syzygium aromaticum*-water extract, exhibiting sizes from 20 to 60 nm, with an average size of 45 nm^29^. Additionally, *Bryophyllum pinnatum* water extract was utilized to fabricate nano-Ag spheres with a size of 54.2 nm^52^. The examination of these factors is crucial before incorporating NPs into various applications due to their physicochemical characteristics, such as reactivity and surface area, which primarily depend on these factors. For example, spherical Ag-NPs measuring 45, 47, and 115 nm were produced using plant extracts from *Brillantaisia patula, Crossopteryx febrifuga*, and *Senna siamea* to inhibit the growth of *E. coli*, *Pseudomonas aeruginosa*, and *Staphylococcus aureus*^53^. The authors noted that *B. patula*-Ag-NPs displayed significant activity, followed by *C. febrifuga*-Ag-NPs and *S. siamea*-Ag-NPs, based on the smaller NP dimensions and varied capping agents related to the specific plant used. Furthermore, Ag-NPs produced from *Syzygium aromaticum* with a dimension of 45 nm and those created from *Laurus nobilis* measuring 12 nm exhibited different antibacterial and antibiofilm activities based on their sizes^29^. The authors found that the nano-Ag with a size of 12 nm demonstrated the largest clear zone in the ranges of 26–48 nm, with an MIC value between 16 and 32 ppm, while the nanostructure of 45 nm displayed inhibition zones of 14–25 nm with an MIC value of 64 ppm. Moreover, the smaller nano-Ag size resulted in a biofilm inhibition percentage of 88%, compared to 70% for the larger nanostructure. Notably, as the effectiveness varies according to NP sizes, it also changes based on their shapes. For instance, spherical Ag-NPs inhibited pathogenic Gram-positive and Gram-negative bacteria at higher percentages compared to the nanorod structure^54^.

**Fig. 5:**
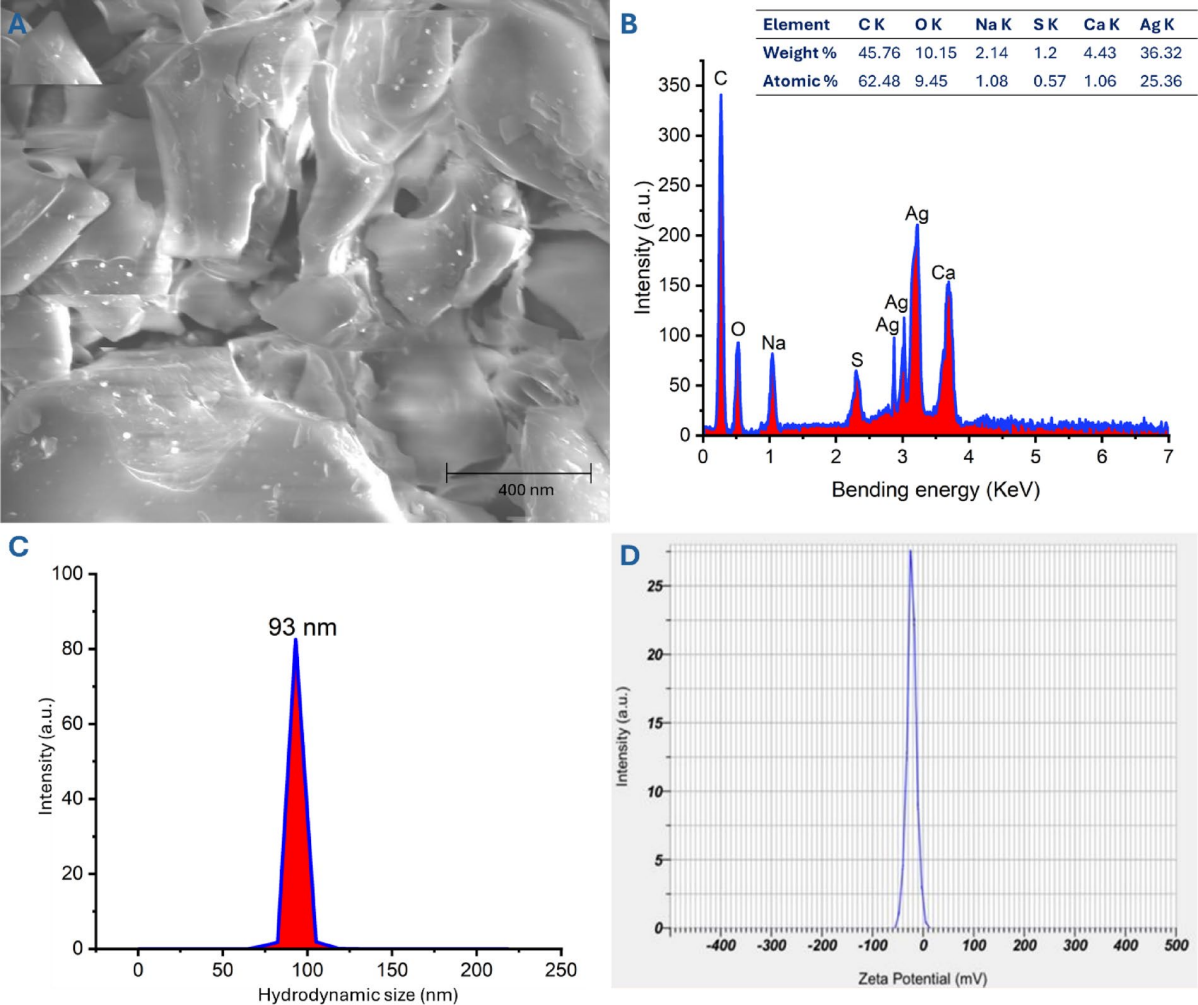
(**A**) SEM image illustrating the aggregation and surface structure of synthesized Ag-NPs, (**B**) EDX analysis revealing the metal constituents, (**C**) DLS for measuring hydrodynamic sizes in the colloidal solution, and (**D**) ζ-potential analysis for assessing surface charge.

The surface structure and particle sizes of the created Ag-NPs were assessed through SEM analysis (Fig. 5A). The SEM image validated the presence of spherical Ag-NPs without any agglomeration. Similarly, the SEM image illustrated the spherical configuration of nano-Ag produced through *Curcuma longa*, with sizes ranging from 10 to 40 nm^55^. Data obtained by Ansari and coauthors indicated that the nano-Ag generated from neem leaf extract tended to exhibit spherical and cubic shapes, with some agglomeration and sizes under 30 nm, as evidenced by SEM analysis^12^.

EDX analysis is an effective method for identifying various ions within biogenic nanostructures. The peaks observed in the energy range of 2.9–3.2 keV correspond to Ag-SPR and signify the successful conversion of Ag^+^ to elemental silver. The present data aligned with previously published studies^37,56,57^. An EDX analysis revealed that the Ag ion in the sample was indicated by substantial weight percentages of 36.3% and atomic percentages of 25.3% (Fig. 5B). The detection of other ions like C, O, Na, S, and Ca with different weight and atomic percentages may be associated with plant capping agents that were dispersed during the EDX analysis, resulting in these peaks^12,58^. In another study, peaks of N, Cl, and O with percentages of 4.9, 7.6, and 11.9%, respectively, were detected alongside the Ag ion in the Ag-NP sample derived from the water extract of *Achillea wilhelmsii*^55^. Furthermore, the weight and atomic percentages of Ag, O, and Cl ions were (83.9, 4.0, and 2.4%) and (64.0, 28.0, and 2.4%), respectively, during the EDX analysis of the nano-Ag sample generated from the leaf extract of *Podocarpus macrophyllus*^59^.

In terms of other characterization techniques, DLS and zeta or electrokinetic potential are vital parameters for measuring the size of NPs and their surface charges in liquids. According to the DLS size distribution graph, the average hydrodynamic sizes in the solution were 93.0 nm (Fig. 5C), which is larger than the sizes obtained from the TEM and XRD analyses. This observation can be attributed to the plant capping agents that contribute to the DLS calculation. Additionally, DLS assesses the hydrodynamic diameter (particle in a hydrated state), unlike TEM, which measures the diameter of solid particles. Furthermore, the agglomeration or uneven distribution of NPs in the solution resulted in larger sizes during measurement^60^. In comparable research, the size of Ag-NPs derived from the leaf water extract of *Pedalium murex* was reported as 73 nm based on the DLS graph, whereas the size was noted as 50 nm from the TEM image^61^.

The uniformity of NPs was assessed based on the polydispersity index (PI), calculated from the DLS analysis. The PI range is from 0 to 1; particles in the solution are regarded as homogeneous if the PI value is under 0.456. In this study, the PI was 0.311, indicating the homogeneity and uniformity of Ag in the colloidal solution. Likewise, the PI of green-synthesized Ag-NPs using extracts from *Ocimum gratissimum*, *Ocimum americanum*, and *Ocimum tenuiflorum* were recorded at 0.207, 0.387, and 0.356, respectively, suggesting the uniformity of particles within a colloidal solution^5^.

The electrokinetic (ζ) potential reflects the stability of NPs based on their surface charge, which directly impacts biomedical and biotechnological applications. The stability level of the nano-Ag structure was categorized according to the ζ value as follows: highly stable in the ranges of ± 20 – ± 30 mV, moderately stable at a ζ value of ± 10 – ± 20 mV, and unstable at ranges of ± 0 – ± 10 mV 57. In this study, the ζ value of rosemary-mediated nano-Ag was − 22.6 mV (Fig. 5D), signifying its high stability in the solution. Likewise, the ζ value for *Urtica dioica* Ag-NP was -24. 1 mV, while those produced by *Potentilla fulgens* exhibited a ζ value of − 18 mV 19. Additionally, Ag-NPs created using leaf water extract of *Urtica dioica* displayed a high negative ζ value of -24.1 mV, indicating significant stability and considerable polydispersity^62^. Interestingly, because of the presence of negative charge on the surface of the nanostructure, the particles are kept separate from one another due to electrostatic repulsion, thus preventing aggregation.

**Fig. 6:**
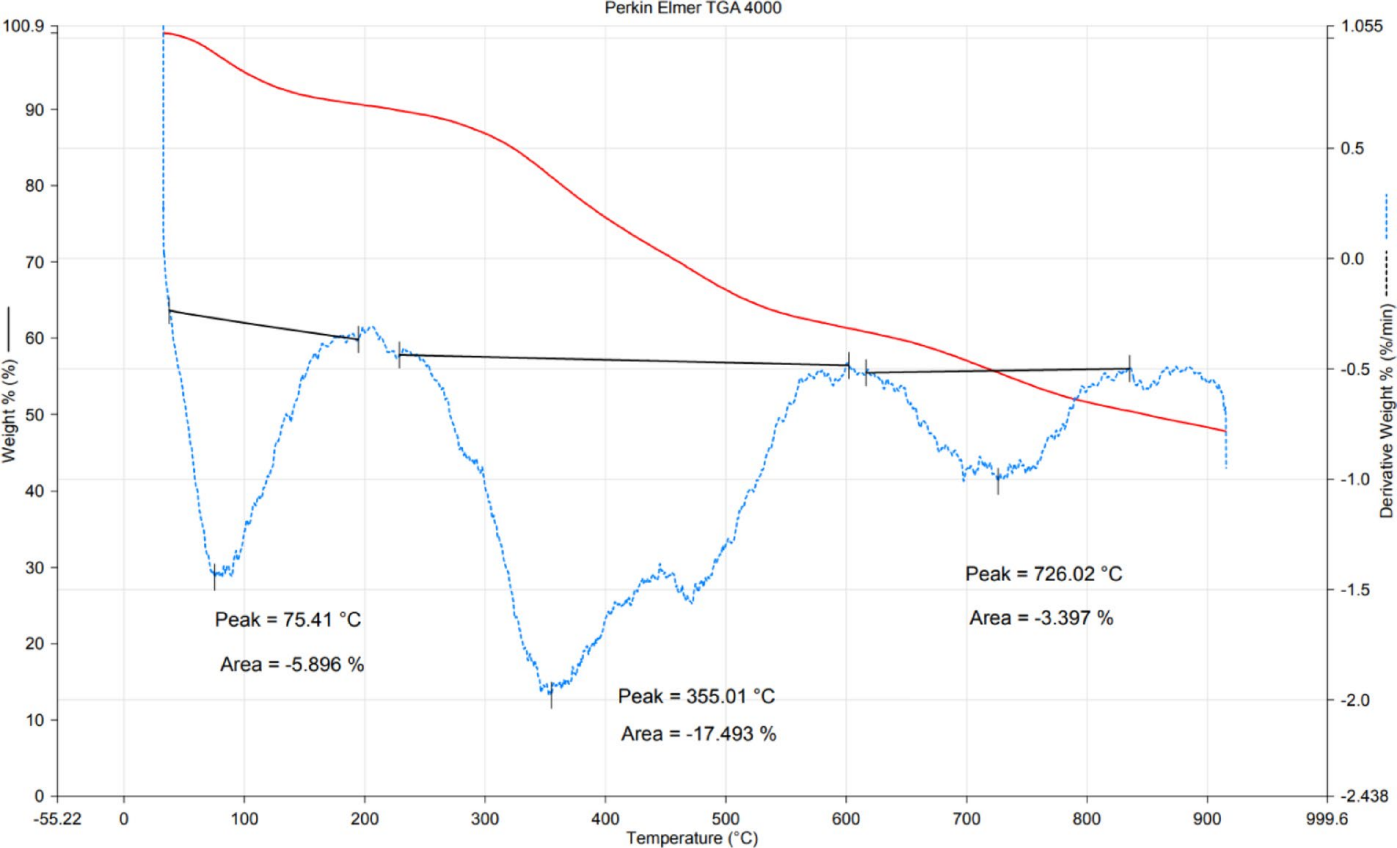
TGA assessment of the fabricated Ag-NPs for evaluation of thermal stability.

Finally, the thermal stability of plant-synthesized Ag-NPs was evaluated using TGA based on relative weight after exposure to heat in the ranges of 30–900 °C (Fig. 6). The TGA curve was divided into phases according to temperature degree and weight loss of materials^63^. Herein, the Ag-NP-TGA curve divided into three phases, the initial phase demonstrates weight loss in the Ag-NPs as temperature rises up to 200 °C with a percentage of 10% due to the evaporation of water present on the NP’s surface. In the second phase, the weight loss amounts to 33% as a result of the degradation of phytochemicals that coat the NP surfaces. Finally, minor weight loss was noted at temperatures up to 600 °C. Furthermore, the DTG curve is categorized into three peaks at temperature levels of 75.4, 355.0, and 726.0 °C with area percentages of − 5.89, − 17.4, and − 3.39%, respectively (Fig. 6). The outcomes were consistent with TGA analysis of Ag-NPs synthesized using the plant extract of *Smyrnium cordifolium*^64^. The authors noted that the initial weight loss occurring before 100 °C was attributed to the removal of H_2_O absorbed on the NP’s surface, while weight loss at temperatures below 690 °C is attributed to the degradation of coating and stabilizing agents. Furthermore, Nassem and colleagues divided the TGA curve of Ag-NPs produced from the peel extract of *Citrus paradisi* into three phases based on the relative temperature degree and weight loss^65^. The authors reported that the first weight loss (up to 100 °C) was due to water removal, while the significant loss occurred at 450 °C as a result of degradation of the plant extract that coated the surface of Ag-NPs.

### Antibacterial activity of the green synthesized Ag-NPs

**Fig. 7:**
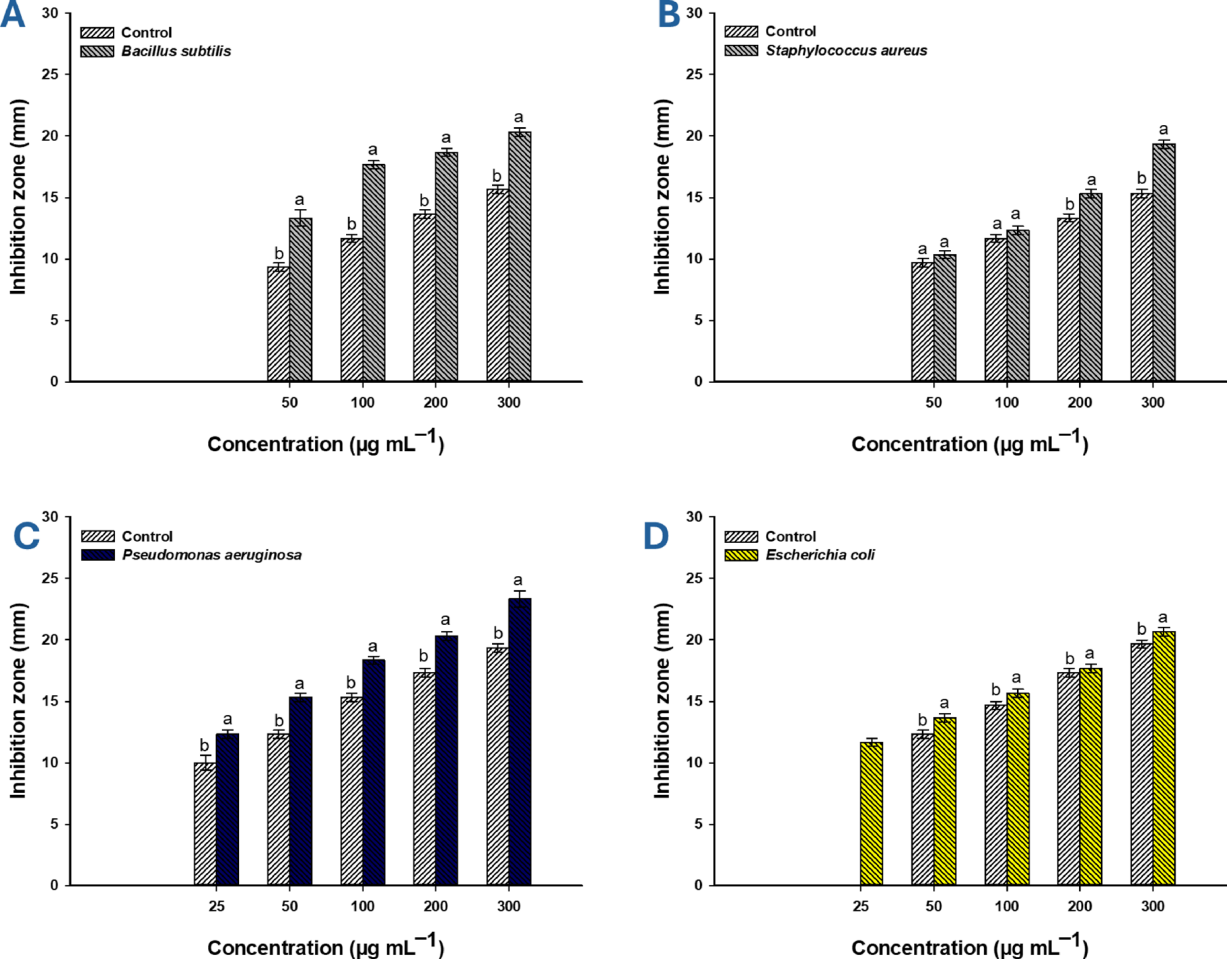
Antibacterial activity of rosemary-mediated biogenic nano-Ag at varying concentrations against designated G+ve and G−ve bacterial strains. Different letters (a and b) on the bars for the same nano-Ag concentration denote significantly different data (*p* ≤ 0. 05).

**Fig. 8:**
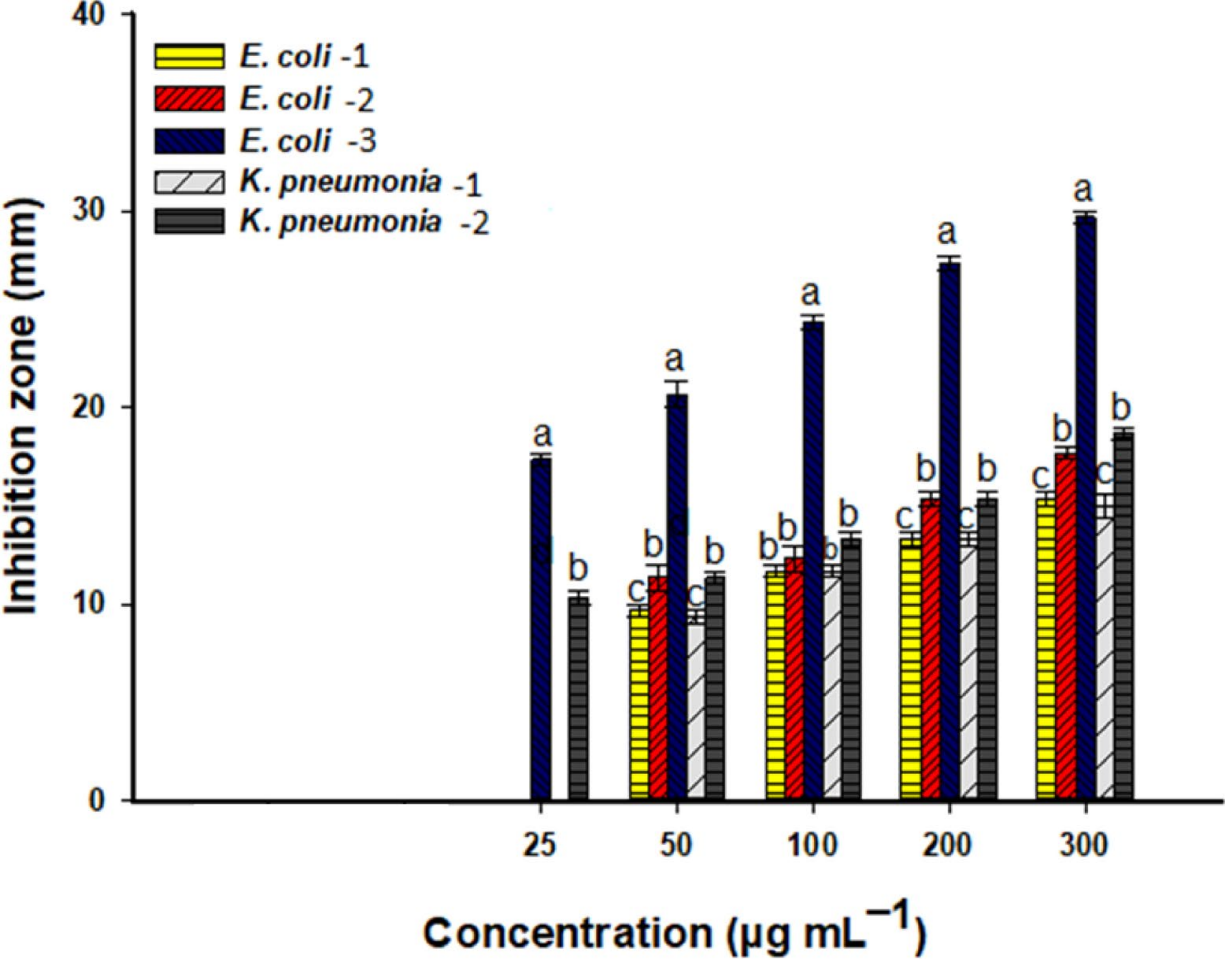
The effectiveness of biogenic nano-Ag against MDR strains, *E. coli*-1 (ESBL, isolated from urine), *E. coli*-2 (ESBL, isolated from sputum), *E. coli*-3 (ESBL, isolated from a wound), *K. pneumonia*-1 (XDR, isolated from a wound), and *K. pneumonia*-2 (ESBL, isolated from urine). Different letters (a, b, and c) on the bars at the same nano-Ag concentration signify significant differences in the data (*p* ≤ 0.05).

The antibacterial effect of rosemary-Ag-NPs was evaluated against nine pathogenic bacterial strains, comprising Gram-positive (G+ve) and Gram-negative (G−ve) multidrug-resistant (MDR) and coded test organisms, which exhibited considerable susceptibility to NPs (Figs. 7, 8). Statistical data analysis revealed that the effectiveness of plant-synthesized nano-Ag against all examined strains was concentration dependent, with the inhibition value increasing at higher nano-Ag concentrations and decreasing at lower levels. The results were consistent with previously published studies. For example, the nano-Ag produced from the water extract of *Crossopteryx febrifuga*, *Brillantaisia patula*, and *Senna siamea* exhibited antibacterial activity against G+ve (*S. aureus*) and G−ve (*E. coli* and *P. aeruginosa*) with a concentration-dependent pattern^53^. Additionally, the antimicrobial activity of Ag-NPs generated from the plant extract of *Neurada procumbens* against G+ve, G−ve, and multicellular fungi was also concentration-dependent^56^. Regarding the coded test bacterial strains, the largest inhibition zone (IZ) was observed at 300 µg mL^–1^ with measurements of 20.3 ± 0.6 for *B. subtilis*, 19.3 ± 0.5 for *S. aureus*, 23.3 ± 1.2 for *P. aeruginosa*, and 20.7 ± 0.6 mm for *E. coli* (Fig. 7A–D). Interestingly, these IZs were higher in comparison to the positive control (Gentamycin), which achieved values of 15.7 ± 0.5, 15.3 ± 0.5, 19.3 ± 0.6, and 19.7 ± 0.6 mm against the same bacterial strains listed above. Recently, the biogenic produced nano-silver mediated by *Portulaca oleracea* yielded maximum IZs of 15.3 ± 0.5 mm against *B. subtilis*, 17.4 ± 1.02 mm for *S. aureus*, 18.7 ± 0.7 mm for *E. coli*, 20.7 ± 0.5 mm for *P. aeruginosa*, 16.3 ± 0.5 mm against *Candida albicans*, and 15.5 ± 0.6 mm for *Aspergillus brasiliensis* at the highest tested concentration (100 µg mL^–1^)^66^. The IZs resulting from the treatment of bacterial strains *B. subtilis*, *S. aureus*, *P. aeruginosa*, and *E. coli* with 100 µg mL^–1^ of synthesized nano-Ag decreased to 17.7 ± 0.6, 12.3 ± 0.5, 18.3 ± 0.6, and 15.7 ± 0.6 mm, respectively, compared to the IZs of the positive control (gentamycin), which were 11.6 ± 0.5, 11.5 ± 0.5, 15.3 ± 0.5, and 14.7 ± 0.6 mm (Fig. 7A–D). As displayed, the activity of nano-Ag was significantly different from that of the positive control activity. Similarly, the biogenic nano-Ag demonstrated high antibacterial efficacy against G + ve strains (*S. aureus* and *B. subtilis*) and G-ve strains (*Klebsiella pneumoniae* and *Proteus vulgaris*), resulting in maximum IZs at 250 µg mL^–1^ and minimum IZs at 100 µg mL^–1^^67^. Recently, the Ag-NPs formed by root extract of *Valeriana jatamansi* showed concentration-dependent antibacterial activity with IZs of (11.2 mm and 18.3 mm), (13.2 mm and 15.7 mm), and (13.6 mm and 16.2mm) at concentrations of 50 and 100 µg mL^–1^ for *E. coli, S. aureus*, and *Streptococcus mutans* respectively^68^. Polymeric NPs, bovine serum albumin covered by amentoflavone and synthesized by leaf extract of *Cassia fistula* showed antibacterial activity against *S. aureus*, *Bacillus cereus,* and *K. pneumoniae* with IZs of 7.3, 6.2, and 7.3 mm respectively^69^.

Variance analysis indicates that G-ve bacteria are more susceptible to nano-Ag than G+ve strains due to differences in cell wall structure, which includes several layers of peptidoglycan in G+ve that impede or delay the penetration of active substances compared to the G−ve cell wall^70^. This observation was consistent with various reported studies indicating that the susceptibility of G-ve bacteria to green synthesized Ag-NPs is greater compared to G+ve strains^71,72^. In terms of MIC determination, it was demonstrated that nano-Ag exhibited MIC against coded bacterial strains of 50 µg mL^–1^ for G+ve strains with IZs ranging from 10 to 13 mm and 25 µg mL^–1^ for G-ve with IZs of 11–12 mm (Fig. 7A–D). Similarly, the MIC of Ag-NPs against *E. coli*, *P. aeruginosa*, and *S. aureus* was (9. 4, 18. 8, and 75 µg mL^–1^), (15, 75, and 300 µg mL^–1^), and (75, 150, and 37. 5 µg mL^–1^) for those synthesized from the water extract of *B. patula*, *C. febrifuga*, and *S siamea*, respectively^53^.

Regarding the MDR susceptibility, the biosynthesis of Ag-NPs mediated by rosemary extract demonstrated significant activity against all examined MDR bacteria in a concentration-dependent manner (Fig. 8). At a high concentration (300 µg mL^–1^), Ag-NPs produced IZs of 15.3 ± 0.6, 17.7 ± 0.5, 29.7 ± 0.6, 15.0 ± 1.0, and 18.7 ± 0.6 mm against *E. coli*-1, *E. coli*-2, *E. coli*-3, *K. pneumonia*-1, and *K. pneumonia*-2, respectively. These IZs decreased to 11.7 ± 0.6, 12.3 ± 1.2, 24.3 ± 0.6, 11.7 ± 0.6, and 13.3 ± 0.6 mm against the same previously mentioned pathogenic MDR strains at 100 µg mL^–1^ (Fig. 8). Here, the MIC of biogenic Ag-NPs against MDR strains, *E. coli*-1, *E. coli*-2, and *K. pneumonia*-1 (XDR), was 50 µg mL^–1^, while it was 25 µg mL^–1^ for the MDR strain *E. coli*-3 that was obtained from wounds and *K. pneumonia*-2 (ESBL). Recently, Ag-nanostructures produced from extracts of *Laurus nobilis* and *Syzygium* aromaticum exhibited promising antibacterial activity against MDR bacterial strains *Staphylococcus lentus*, *Staphylococcus sciuri*, and *Kocuria rosea*^29^. The authors demonstrated that the IZs formed using 50, 100, and 150 µg mL^–1^ of Ag-NPs derived from *L. nobilis* against *K. rosea*, *S. sciuri*, and *S. lentus* were (32, 40, and 48 mm), (26, 27, and 30 mm), and (28, 30, and 32 mm), respectively, whereas the IZs formed upon treatment with Ag-NPs produced by *S. aromaticum* were (15, 20, 25 mm), (14, 17, 20 mm), and (17, 20, and 22 mm), respectively. Furthermore, the authors reported that the MIC for Ag-NPs synthesized using *L. nobilis* ranged from 16 to 32 µg mL^–1^, while it was 64 µg mL^–1^ for Ag formed by *S. aromaticum* against three tested MDR bacterial strains. The efficacy of biogenic Ag nanostructures against coded pathogenic and MDR strains may be attributed to their smaller sizes. Ali and coauthors indicated that the effectiveness of green synthesized nano-Ag with a size of 12 nm was greater against MDR strains, *Staphylococcus lentus*, *Staphylococcus sciuri*, and *Kocuria rosea*, in contrast to a size of 45 nm^29^. Smaller NP sizes are notable for their high surface area relative to volume, promoting contact with pathogenic microbial cells, which elevates the release of toxic Ag^+^ ions. These harmful ions interfere with DNA replication, protein function, cell wall integrity, and other cellular processes^73^. The primary resistance mechanisms found in MDR include alterations in membrane permeability, the presence of efflux pumps, and modifications in enzymatic activities. All of these factors allow them to withstand antibiotic effects. Consequently, the smaller sizes of NPs and their heightened reactivity can counteract the mechanisms of MDR resistance^29^.

The antibacterial actions of Ag-NPs can be outlined within the following categories: generation of ROS, increased production of free radicals from the Ag-NPs surface, release of toxic Ag^+^ ions, reduction in ATP levels due to reactions with bacterial cells, damage to respiratory enzymes, disruption of DNA replication, and interference with the functions of macromolecules (proteins, amino acids, and ribosomes) inside the cells^74,75^. Additionally, Ag-NPs can disrupt the integrity of the cytoplasmic membrane. The electrostatic attraction between Ag-NPs and the phospholipid bilayer results in destabilization and alters the cell membrane’s fluidity and permeability^33^. Moreover, the binding of Ag-NPs to cell membranes results in the creation of new pores, facilitating the leakage of intracellular components vital for bacterial growth^1^. Furthermore, the release of toxic Ag^+^ ions inside the cells increases oxidative stress and leads to lipid peroxidation, a loss of membrane integrity, damage to membrane-associated proteins and enzymes, and a breakdown of selective permeability functions^30^. Also, the toxic Ag+ inside the bacterial cells can be reacting with amino acids-SH group forming Ag-SH causing protein coagulation and amino acid damage^76^.

### Antidiabetic activity of the green synthesized Ag-NPs

**Fig. 9:**
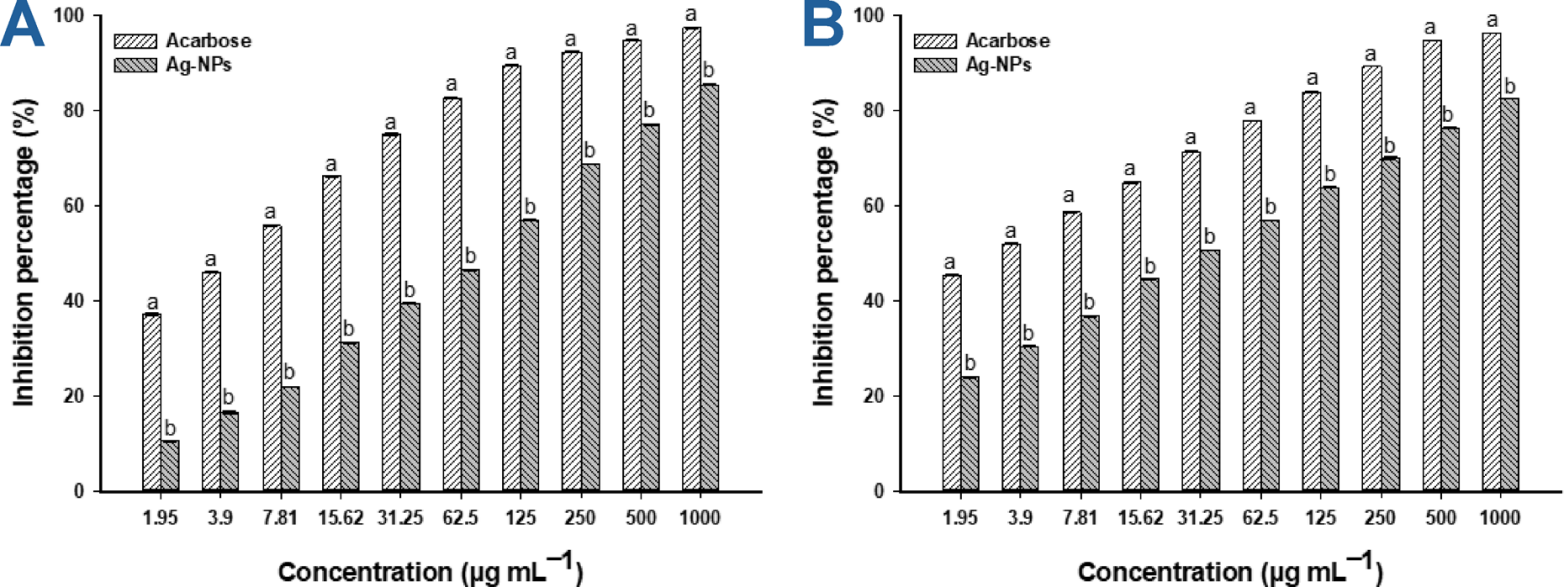
Antidiabetic effect of rosemary-mediated biogenic Ag-NPs. (**A**) indicates the inhibition percentage of the α-amylase enzyme; (**B**) shows the inhibition % of the α-glucosidase enzyme after treatment with nanostructures at different concentrations. Different letters (a and b) on the bars at the same concentration indicate the data are significantly different (*p* ≤ 0.05).

Α-amylase and α-glucosidase enzymes play an essential role in carbohydrate breakdown and glucose absorption. These enzymes break down polysaccharides, like starch, into disaccharides such as dextrin and maltose, followed by further breakdown into glucose, thereby controlling blood glucose absorption^30^. Various concentrations of plant-synthesized Ag-NPs were utilized to determine the inhibition percentages of α-amylase and α-glucosidase compared to the positive control (acarbose) (Fig. 9A,B). Statistical analysis revealed that the inhibition percentages of the two enzymes were dependent on the Ag-NP concentration, increasing with higher NP concentration and decreasing with lower concentration. This observation aligns with findings from Balu et al., who reported that the inhibition percentages of α-amylase and α-glucosidase due to plant-synthesized Ag-NPs were concentration-dependent; the inhibition rose as concentration increased from 10 to 100 µg mL^–1^^30^. In this study, the highest inhibition percentages for α-amylase were achieved at 1000 µg mL^–1^, yielding a value of 85.5 ± 0.2% compared to acarbose’s 97.5 ± 0.2% (Fig. 9A). Similarly, the inhibition of α-glucosidase was achieved with percentages of 82.6 ± 0.2% versus 96.3 ± 0.2% of the positive control at the same maximum concentration (Fig. 9B). The inhibition percentages declined as the Ag-NPs concentration reduced, recording values of (77.3 ± 0.3, 57.1 ± 0.2, 39.6 ± 0.3, 21.9 ± 0.1, and 10.6 ± 0.3%) for α-amylase compared to positive control inhibition percentages of (94.9 ± 0.2, 89.5 ± 0.2, 75.1 ± 0.5, 55.9 ± 0.3, and 37.3 ± 0.2%) at 500, 125, 31.25, 7.81, and 1.95 µg mL^–1^ of nano-Ag, respectively (Fig. 9A). Likewise, the α-glucosidase inhibition percentages were (76.5 ± 0.1, 63.9 ± 0.3, 50.8 ± 0.1, 36.8 ± 0.3, and 24.1 ± 0.2%) versus acarbose inhibition percentages of (94.8 ± 0.1, 83.9 ± 0.2, 71.3 ± 0.1, 58.7 ± 0.2, and 45.5 ± 0.1%) at the same concentrations mentioned above, respectively (Fig. 9B). Recently, Ag-NPs derived from the root water extract of the medicinal plant *Martynia annua* demonstrated antidiabetic effects through α-amylase inhibition, with percentages of 35.9, 44.6, 61.1, 66.1, and 78.4% at concentrations from 50 to 250 µg mL^–1^, with an IC_50_ value of 120.1 µg mL^–1^^77^.

The IC_50_ value (the concentration of nano-Ag that inhibits 50% of enzyme activity) was calculated for both the NPs and the positive control. Data analysis indicated that the IC_50_ value of nano-Ag was 139.5 ± 9.6 µg mL^–1^ for α-amylase (versus 8.9 ± 0.5 µg mL^–1^ for acarbose) and 7.4 ± 1.3 µg mL^–1^ for α-glucosidase compared to 15.3 ± 0.5 µg mL^–1^ for acarbose. Here, the α-glucosidase enzyme exhibited greater sensitivity to green-synthesized nano-Ag in comparison to the α-amylase enzyme. This finding was consistent with results reported by Govindappa et al.,^78^, which showed that Ag-NPs produced from *Calophyllum tomentosum* water extract had higher activity against α-glucosidase inhibition, recording 50% at a concentration of 500 µg mL^–1^, as opposed to 20% inhibition of α-amylase at the same concentration. Additionally, the leaf extract of *Punica granatum* was employed to produce nano-Ag with strong potential to inhibit α-amylase and α-glucosidase enzymes^79^. The authors noted that the IC_50_ values for the synthesized nanostructures were 65.2 and 53.7 µg mL^–1^ for α-amylase and α-glucosidase, respectively. Notably, Balu and colleagues reported that the IC_50_ values for Ag-NPs synthesized using acetone, ethanol, and water petal extracts of *Rosa indica* were identical for both enzymes, recorded at 50, 50, and 75 µg mL^–1^, respectively^30^. The increased activity of Ag-NPs against α-glucosidase relative to α-amylase could be attributed to the smaller and more compact structure of α-glucosidase, facilitating Ag-NP attachment to its active sites, thereby inactivating these sites or interacting with amino acid residues and disrupting its function. Conversely, α-amylase has a larger and more complex structure, making it less effective for Ag-NPs to bind to its active sites^80^. Furthermore, NPs can engage directly with α-glucosidase through functional groups (e.g. -SH), which are crucial for its function, while the rigid structure of α-amylase hinders the interaction between its functional groups and NPs, leading to reduced impact^81^. Additionally, the compositions and mechanisms of action of enzymes significantly influence their varying susceptibility to NPs. α-glucosidase acts on oligosaccharides, hydrolyzing them into monosaccharides, resulting in greater susceptibility to disruption by NPs. In contrast, α-amylase targets large polysaccharides, which complicates interactions with NPs at its active sites^82^.

Ag-NPs, particularly those produced using plant extracts, are distinguished by their effectiveness in combating diabetes through various mechanisms. For example, Ag-NPs along with their plant capping agents, including flavonoids and phenolic compounds, can enhance the phosphorylation process of the insulin receptor substrate, which results in improved glucose absorption and decreased hyperglycemia^79^. Additionally, due to the antioxidant properties of Ag-NPs, they have the capability to eliminate harmful free radicals, which contributes to the diminishment of oxidative stress, a key factor for diabetics. Ag-NPs generated from plant extracts can regenerate and safeguard pancreatic β-cells, which play a crucial role in insulin secretion. Furthermore, the plant capping agent can support this function^83^. Following nano-Ag treatment, the uptake of glucose by muscle and adipose tissue may be enhanced due to their effectiveness in elevating GLUT4 expression, which results in a reduction in hyperglycemia^84^.

### Antioxidant activity of the green synthesized Ag-NPs

**Fig. 10:**
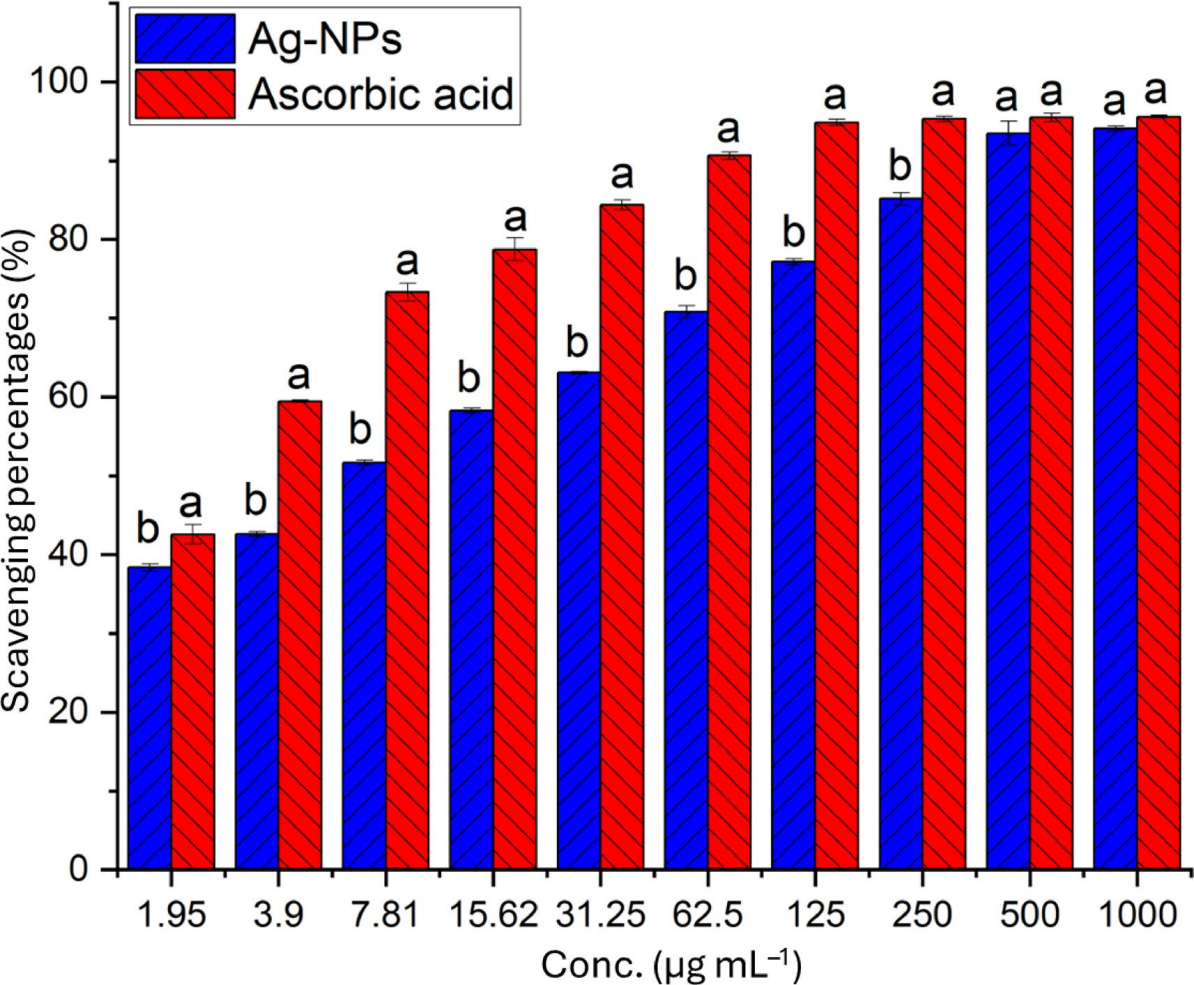
DPPH scavenging assay comparing plant-mediated biogenic nano-Ag *versus* ascorbic acid as a positive control. Different letters (a and b) on the bar at the same concentration refer to significantly different data (*p* ≤ 0.05).

The primary issue in the healthcare field is the diseases triggered by free radicals generated within the cells, including heart disease, neurological disorders, vascular diseases, cancer, diabetes, kidney failure, etc. These free radicals adversely affect proteins, nucleic acids, amino acids, and essential micro- and macromolecules inside the cells^85^. Consequently, finding new substances with antioxidant properties is the principal objective of research. Antioxidant substances play a crucial role in neutralizing or reducing the oxidative damage caused by these free radicals by donating electrons and converting them into harmless substances^25,86^. The DPPH assay is the most widely utilized method for assessing the potential of new active substances as antioxidant agents^78^. Therefore, this study examines the antioxidant activity of Ag-NPs in comparison to a positive control (ascorbic acid). The results indicated that Ag-NPs exhibit promising antioxidant activity in a concentration-dependent manner^78^. At the highest concentrations (1000 and 500 µg mL^–1^), no significant difference was observed between the antioxidant capacity of Ag-NPs and ascorbic acid; the scavenging percentages were 94.1 ± 0.3 and 92.5 ± 1.5% for Ag-NPs compared to 95.6 ± 0.2 and 95.4 ± 0.03% for ascorbic acid (Fig. 10). Recently, biogenic Ag-NPs demonstrated a scavenging % of 88.5 and 84.5 at the maximum tested concentrations (200 and 100 µg mL^–1^, respectively) when compared to ascorbic acid percentages (89.3 and 85.1%)^66^. The scavenging percentages decreased with lower concentrations. For example, biogenic Ag-NPs showed scavenging percentages of (77.2 ± 0.4, 63.1 ± 0.2, 51.7 ± 0.3, and 38.4 ± 0.2%) at concentrations of 125, 31.25, 7.81, and 1. 95 µg mL^–1^, respectively, in comparison to standard percentages (94.9 ± 0.4, 84.4 ± 0.6, 73.3 ± 1.1, and 42.6 ± 1.2%) at the same concentrations (Fig. 10). Likewise, the Ag-NPs synthesized from water extract of *Atrocarpus altilis* leaves demonstrated 79.7% scavenging at 100 µg mL^–1^^87^.

Effective concentration (EC_50_) refers to the concentration of nano-Ag that scavenges 50% of free radicals. In this study, the EC_50_ of rosemary synthesized Ag-NPs is low, measuring 7.81 µg mL^–1^ compared to 3.27 µg mL^–1^ for ascorbic acid. In a different study, the EC_50_ of *Psidium guajava*-Ag-NPs was found to be 52.5 µg mL^–1^ in comparison to 25.5 µg mL^–1^ for ascorbic acid^88^, which was higher than the obtained results. Additionally, the EC_50_ of Ag-NPs derived from *Erythrina suberosa* was noted to be 30.1 µg mL^–1^^89^, while those produced by *Zea mays* showed an EC_50_ of 385.9 µg mL^–1^^90^. Also, the EC_50_ of Ag-NPs mediated by leaf aqueous extract of *Pterolobium hexapetalum* was 114.9 µg mL^–1^^91^. As shown, the low EC_50_ value in the current study compared to published ones indicates the high activity of *R. officinalis-*Ag-NPs*.* The differences in scavenging activity and EC_50_ values could be due to the capping agents, which varied among the plant species employed for synthesis.

The structure of nano-silver has the capability to neutralize the harmful effects of free radicals, such as H_2_O_2_ and ^•^OH, subsequently diminishing oxidative stress. Additionally, certain antioxidant enzymes such as superoxide dismutase, catalase, and peroxidase can be upregulated through interaction with Ag-NPs^17^. Furthermore, some metal ions that induce oxidative stress can be chelated by nano-Ag^92^. Alongside bioactive compounds from plant species that function as capping agents, they can enhance the antioxidant capacity of the NPs^51^. Interestingly, Ag-NPs can mitigate some harmful effects caused by ROS, such as preventing lipid peroxidation in cell membranes, inhibiting specific inflammatory pathways, and hindering the oxidation of proteins and DNA^17^.

### Anticancer activity of the green synthesized Ag-NPs

**Fig. 11:**
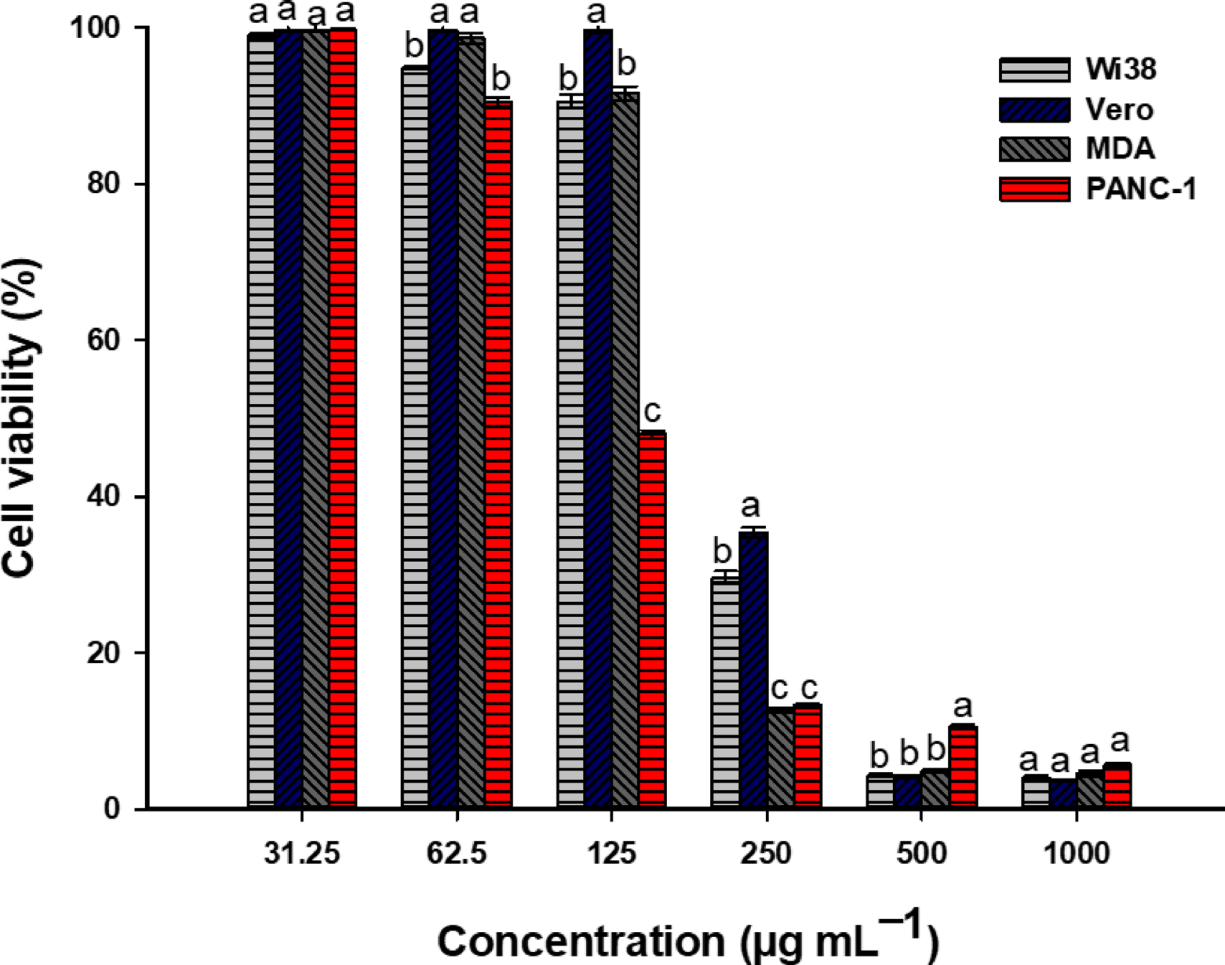
In vitro anticancer and biocompatibility assessment of plant-mediated Ag-NPs against cancer and normal cell lines via MTT assay technique. Different letters (a, b, and c) for the same concentration indicate the data are significantly different (*p* ≤ 0. 05).

The most efficient method for evaluating the potential harmful effects of various compounds, including drugs, chemicals, and environmental pollutants, on living cells is through cell-based toxicological tests^93^. Additionally, the colorimetric MTT assay is a dependable and sensitive method for measuring cell growth and toxicity based on the metabolic activity within living cell mitochondria^94^. In this study, we conducted MTT assays using the prepared phyto-derived silver NPs at concentrations ranging from 1000 to 31.25 μg mL^–1^ to assess their toxicological effects against a variety of normal cell lines, specifically Wi38 (human lung fibroblasts) and Vero (monkey kidney epithelial cells), as well as cancerous cell lines, namely MDA (human breast adenocarcinoma) and PANC-1 (human pancreatic epithelioid carcinoma) (Fig. 11). The results of the analysis confirmed the promising characteristics of green Ag-NPs as selective anticancer agents in a concentration-dependent manner. Moreover, the treated normal cells (Wi38 and Vero) experienced less impact from the applied Ag-NP concentrations. As illustrated, the cell viability of both normal and cancer cells did not show statistically significant differences (*p* > 0.05) at both high (1000 μg mL^–1^) and low (31.25 μg mL^–1^) concentrations (Fig. 11). Conversely, the cell viability percentages of the cancer cell lines, MDA and PANC-1, were drastically reduced, recording 12.7 ± 0.5 and 13.3 ± 0.4%, respectively, at 250 μg mL^–1^, in comparison to the viability percentages of normal cells (29.7 ± 1.4% for Wi38 and 35.4 ± 1.1% for Vero) at the same concentration (*p* ≤ 0. 05). Upon reducing the concentration to 125 μg mL^–1^, the viability of PANC-1 cells was significantly reduced (47.9 ± 0.8%) when compared with the viability of the other cells (91.5 ± 1.6% for MDA, 90.5 ± 1.5% for Wi38, and 99.7 ± 0.8% for Vero) (Fig. 11). Similarly, the viability of HL-60 cancer cells was 20% following treatment with 2 mM of Ag-NPs produced by *R. officinalis* and incubated for 24 h^95^.

**Fig. 12:**
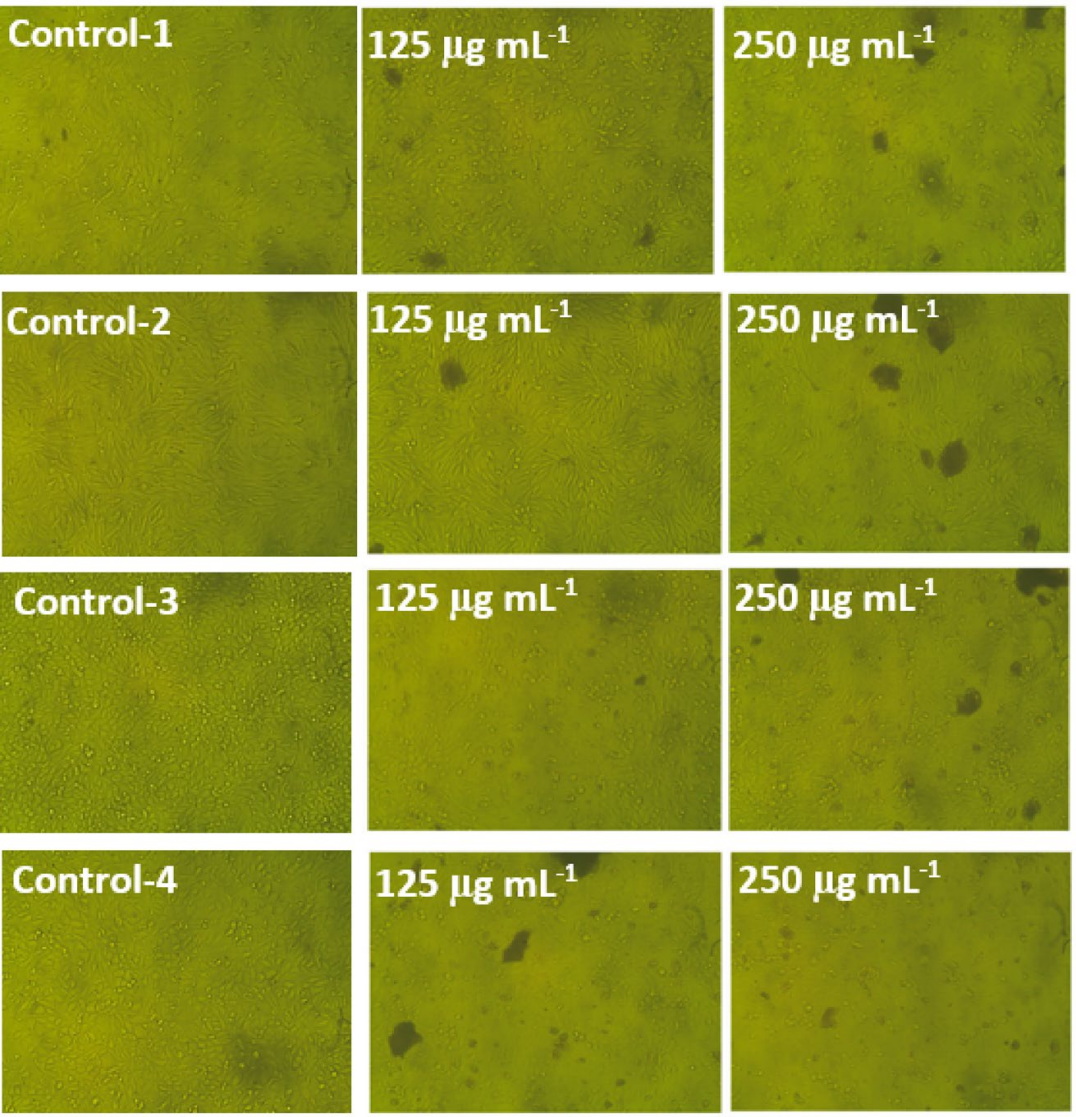
Phase contrast microscopic images illustrating morphological changes in normal and cancerous mammalian cell lines following treatment with green synthesized silver nanoparticles (Ag-NPs) at concentrations of 125 and 250 μg mL⁻^1^. “1” Wi38 (normal human lung fibroblasts), “2” Vero (normal monkey’s kidney epithelial cells), “3” PANC-1 (human pancreatic carcinoma), and “4” MDA (human breast adenocarcinoma).

Because compounds with low IC_50_ values are defined by their specific effectiveness with minimal side effects on normal cells of patients, studies in drug discovery depend on IC_50_ values to evaluate the efficacy of substances tested as potential medications^96^. Interestingly, the IC_50_ values for cancer cells in this study were considerably lower than those for the tested normal cells. The IC_50_ values for MDA and PANC-1 were found to be 177.2 ± 1.76 and 115.3 ± 1.67 μg mL^–1^, respectively. In comparison, normal Vero and Wi38 were determined to have IC_50_ values of 233.0 ± 6.74 and 207.4 ± 2.64 μg mL^–1^, respectively (Fig. 11). This finding supports that 250 μg mL^–1^ of Ag-NPs could be applicable in biomedical fields to achieve greater efficacy against cancer cells while exerting limited effects on healthy cells. In agreement with our findings, biogenic Ag-NPs exhibited significant in vitro antiproliferative activity against Caco-2 (colon cancer) and MCF-7 (breast cancer) human cell lines, with IC_50_ values of 156 and 160 μg mL^–1^, respectively^1^. Similarly, the leaf extract of *Lantana camara* synthesized Ag-NPs demonstrated notable anti-cancer effects against human breast cancer (MCF7) and lung cancer (A549) cell lines, with IC_50_ values of 46.6 and 49.5 μg mL^–1^, respectively^97^. Furthermore, Ag-NPs obtained from actinobacteria have recently shown improved antibacterial properties against *Escherichia coli*, *Pseudomonas aeruginosa*, *Klebsiella pneumoniae*, and *Staphylococcus aureus*. They also significantly reduced the viability of the human breast cancer (MCF-7) and murine macrophage cell line RAW 264.7^98^. Phase contrast microscopy confirmed that green synthesized Ag-NPs induced apoptosis in cancer cells, as evidenced by morphological changes such as shrinkage, rounding, while normal cells showed minimal alterations (Fig. 12). These effects align with previous studies linking such changes to oxidative stress, mitochondrial dysfunction, DNA damage, and apoptotic caspase activation^28^.

Phase contrast microscopy was employed in this study to examine the phenotypic alterations of the evaluated mammalian cells and confirm the toxicity of silver nanoparticles against them; the microscopic analysis indicated that the malignant cells lost the monolayer characteristics of epithelial cells due to cell shrinkage and migration, along with increased granularity, rounding, and cellular distortion. Similarly, when Caco-2 cancer cell lines were subjected to green silver NPs, Salem et al.^99^ noted comparable morphological changes in a concentration-dependent manner.

The anticancer activity might be linked to the generation of reactive oxygen species (ROS), disruption of the nucleus and DNA, changes in mitochondrial membrane potential, inactivation of LDH (lactate dehydrogenase), and activation of apoptotic caspases. In addition, silver nanoparticles disrupt enzymes and proteins that are crucial for the signaling pathways required for the proliferation and survival of cancer cells, rendering them ineffective. Silver nanoparticles also boost the immune response to hinder and eradicate these abnormal cells. The accumulation of nanoparticles inside cells via phagocytosis damages malignant cells by disrupting cellular organelles and functions^24^.

## Conclusion

The metabolites derived from the aqueous extract of *Rosmarinus officinalis* were utilized to create Ag-NPs. various biosynthesis parameters, such as the concentration of AgNO_3_, pH value, contact time, temperature, and plant extract concentration, were modified to achieve optimal yield. The green-synthesized Ag-NPs were characterized through visual observation (yellowish-brown color) and comprehensive analyses, including UV–Vis spectroscopy, FT-IR, XRD, TEM, SEM, EDX, TGA, DLS, and ζ-potential measurements. Several biological activities, including antibacterial activity against MDR, antioxidant potential, antidiabetic effects, and anticancer activity, were assessed for the biosynthesized Ag-NPs, indicating that their effects were concentration-dependent. The produced Ag-NPs effectively inhibited pathogenic bacterial strains, producing inhibition zones (IZs) of 15–29 mm at 300 µg mL⁻^1^. Furthermore, Ag-NPs serve as scavengers for DPPH free radicals, demonstrating significant antioxidant effects. They also exhibited antidiabetic properties, with α-glucosidase being more susceptible to nano-Ag than α-amylase. Interestingly, the synthesized Ag-NPs were more effective against cancer cells at lower concentrations than against normal cell lines. In summary, our findings demonstrate that green-synthesized Ag-NPs possess promising multifunctional biomedical properties. Future research plans will focus on exploring the molecular and genetic mechanisms through which green-synthesized Ag-NPs combat MDR infections, in addition to investigating the potential synergistic effects of Ag-NPs combined with standard antibiotics. Comprehensive in vivo studies will also be conducted. Additionally, by functionalizing Ag-NPs with plant-derived active compounds, future studies will aim to enhance their capacity to target specific cancer cells and operate as a nano-drug delivery system. Finally, we aim to broaden our research into the potential of wound healing by applying green-synthesized Ag-NPs to biopolymers.

## Data Availability

The datasets used and/or analyzed during the current study are available from the corresponding author upon reasonable request.
